# Dermal TRPV1 innervations engage a macrophage and fibroblast containing pathway to activate hair growth in mice

**DOI:** 10.1016/j.devcel.2024.05.019

**Published:** 2024-06-07

**Authors:** Tamar L Ben-Shaanan, Konrad Knöpper, Lihui Duan, Ruiqi Liu, Hanna Taglinao, Ying Xu, Jinping An, Maksim V. Plikus, Jason G Cyster

**Affiliations:** 1. Howard Hughes Medical Institute and Department of Microbiology and Immunology, University of California, San Francisco, San Francisco, CA 94143, USA.; 2. Current Address: State Key Laboratory of Molecular Development Biology, Institute of Genetics and Developmental Biology, Chinese Academy of Sciences, Beijing, China and University of Chinese Academy of Sciences, Beijing, China.; 3. Department of Developmental and Cell Biology, University of California, Irvine, Irvine, CA, USA

## Abstract

Pain, detected by nociceptors, is an integral part of injury, yet whether and how it can impact tissue physiology and recovery remain understudied. Here we applied chemogenetics in mice to locally activate dermal TRPV1 innervations in naïve skin and found it triggered new regenerative cycling by dormant hair follicles (HFs). This was preceded by rapid apoptosis of dermal macrophages, mediated by the neuropeptide calcitonin gene-related peptide (CGRP). TRPV1 activation also triggered a macrophage-dependent induction of osteopontin (Spp1) expressing dermal fibroblasts. The neuropeptide CGRP and extracellular matrix protein Spp1 were required for the nociceptor-triggered hair growth. Finally, we showed that epidermal abrasion injury induced Spp1-expressing dermal fibroblasts and hair growth via a TRPV1 neuron and CGRP dependent mechanism. Collectively, these data demonstrated a role for TRPV1 nociceptors in orchestrating a macrophage and fibroblast-supported mechanism to promote hair growth and enabling the efficient restoration of this mechano- and thermo-protective barrier after wounding.

## eTOC blurb

Ben-Shaanan et al. demonstrate a cellular pathway causally connecting pain mediating cutaneous TRPV1 innervations with accelerated hair growth in naïve skin, as well as hair regrowth after epidermal abrasion. This cellular pathway involves dermal macrophage apoptosis triggered by the neuropeptide CGRP, followed by a macrophage-dependent increase in the frequency of fibroblasts expressing the hair growth promoting factor Spp1.

## Graphical Abstract

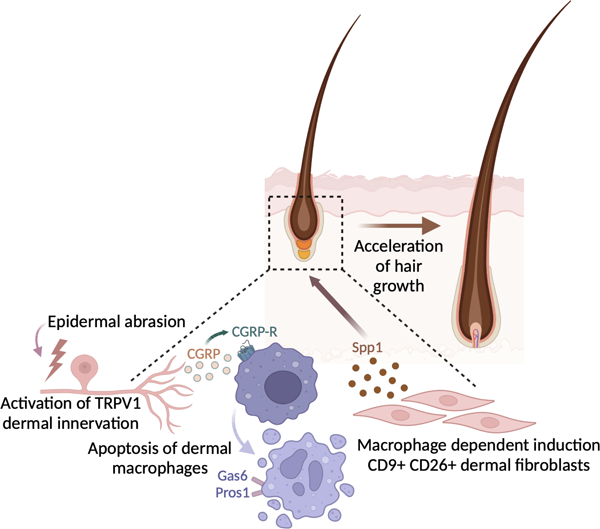

## Introduction

The skin is the largest sensory organ and a major barrier site with a significant regenerative capacity^1–3^. Breach of the skin barrier, due to injury, initiates a process of wound healing that involves an orchestrated response by immune, vascular, stromal, and epithelial cells^4^. Noxious stimuli, which potentially can breach the skin, activate nociceptive neurons that communicate with the central nervous system via the spinal cord to induce pain sensation^5,6^. Pain experienced due to wounding has been linked to specific nocifensive behavioral responses aimed at protecting the wounded tissue and thereby supporting its healing (e.g., aversion to appley pressure on a wounded limb, or licking a wounded skin area to remove debris)^5^. Nevertheless, whether and how pain and its underlying peripheral nociceptors can directly impact tissue recovery is not fully understood.

TRPV1 expressing Aδ- and C-fibers are a subset of peripheral nociceptors activated by vanilloid chemicals, heat, and inflammation^7–9^. TRPV1 expressing C-fibers innervate the dermis and epidermis and are ideally positioned to sense environmental stimuli^10,11^ and directly interact with multiple skin cell types^12^. Recent work highlights the ability of TRPV1 innervations to impact local immune responses^13–17^. Underlying many of these effects are the neuropeptides substance P (SubP) and Calcitonin gene related peptide (CGRP) which are secreted mostly by activated cutaneous TRPV1 innervations. In wounded skin, CGRP has been associated with stimulating angiogenesis^18^ and the proliferation and migration of keratinocytes^19^. However, comprehensive understanding of nociceptors’ roles in orchestrating regenerative responses and their underlaying cellular mechanisms is lacking.

Superficial epidermal wounds, such as abrasions, rely, partly, on hair follicle stem cells (HFSCs) to aid in the replacement of the lost epidermis^20^. HFSCs, supported by specialized stromal cells, sustain the ability of HFs to undergo regenerative cycling throughout life^21,22^. Interestingly, scrapes and bruises that often result in superficial epidermal removal can be quite painful. While sensory skin innervations were shown to affect incision wound recovery^23,24^, their impact on the HFSC niche and associated stromal cells remains unclear.

Here we used chemogenetics (also known as Designer Receptors Exclusively Activated by Designer Drugs or DREADDs) to activate cutaneous TRPV1 nociceptors in mouse dorsal skin and found that it accelerated hair growth. Preceding this phenotype, we observed a rapid reduction in dermal macrophages due to cell death. This was followed by macrophage-dependent and Axl tyrosine kinase-inhibitor sensitive changes in the dermal fibroblast gene expression profile indicative of hair growth support, specifically the expression of the hair growth promoting factor Spp1. Mechanistically, hair growth activation, changes in dermal fibroblast subsets and the reduction in macrophage frequency, were all dependent on CGRP. Using a mouse model for epidermal abrasion with tape stripping, we demonstrate that TRPV1 innervations in this context promote hair regrowth. Also, dermal fibroblasts, which were necessary for hair regrowth after tape stripping, reacted to this type of wounding in a TRPV1-dependent manner. Taken together, our findings demonstrate a crucial role for TRPV1 cutaneous innervations in mediating hair regrowth after epidermal abrasion and define the elements underlying this pathway.

## Results

### Repeated activation of cutaneous TRPV1 dermal innervations induced local inflammation.

Chemogenetics enable the selective manipulation of targeted cell populations and were previously used to manipulate peripheral TRPV1 innervations^25,26^. Neurons expressing the activating DREADD (known as hM3Dq) can be engaged by administering a small molecule ligand, like clozapine-N-oxide (CNO)^27,28^, which leads to increased neuronal firing rate^27,28^. By crossing *TRPV1-Cre* mice with DREADD-floxed mice (*CAG-LSL-Gq-DREADD*) we generated mice with TRPV1 driven expression of hM3Dq (referred to as TRPV1Cre^hM3Dq^). Immunofluorescence staining for the HA-tag (encoded together with DREADD) in the dermis and epidermis of TRPV1^hM3Dq^ mice, revealed staining restricted to neuronal fibers, some resembling free nerve endings (white arrows; figure S1A) or circumferential innervations wrapped around HFs (orange arrows; Figure S1A).

TRPV1 neurons serve as peripheral sensors of heat, and their activation enhances heat sensitivity^29^. To validate the use of chemogenetics to elicit a pain response, we used the hot plate test. Intraplanar injection of CNO to TRPV1Cre^hM3Dq^ mice significantly reduced the response time to heat stimuli, indicative of increased pain sensitivity (Figure S1B). In control mice that do not express DREADDs heat sensitivity was unaltered, and using histology we were unable to detect effects on the skin after CNO injection (Figure S1C).

Repeatedly activating cutaneous TRPV1 innervations with optogenetics was shown to induce inflammation and tissue thickening^16^. To determine the effects of DREADDs activation of the same neuronal subsets, we injected CNO intradermally in the dorsal skin of TRPV1Cre^hM3Dq^ mice and their controls. Three daily TRPV1 activation treatments were sufficient to significantly increase dermal thickness around the injection site (Figure S1D). This repeated cutaneous TRPV1 activation also induced dermal infiltration of neutrophils and monocytes, indicative of an inflammatory response, which was restricted to the CNO-treated area of the skin (Figure S1E and S1F). An increased in *Il6* expression both in the upper dermis and subcutis (Figure S1G) was observed, which is in accord with the response to optogenetic stimulation^16^. Expression of *Tnf* and *Tgfb1* increased only in the subcutis (Figure S1H and S1I). While cutaneous optogenetic TRPV1 activation increased dermal expression of *Il17* and *Il23*^16^, we did not detect analogous expression changes following neuronal activation with DREADDs (Figure S1J). These data show that DREADD-induced TRPV1 activation triggers inflammatory changes similar, but not identical, to the ones triggered by optogenetics, and add to our understanding of the neurogenic inflammatory processes.

### Repeated activation of cutaneous TRPV1 innervations impacts dermal fibroblast subsets.

**Figure 1:**
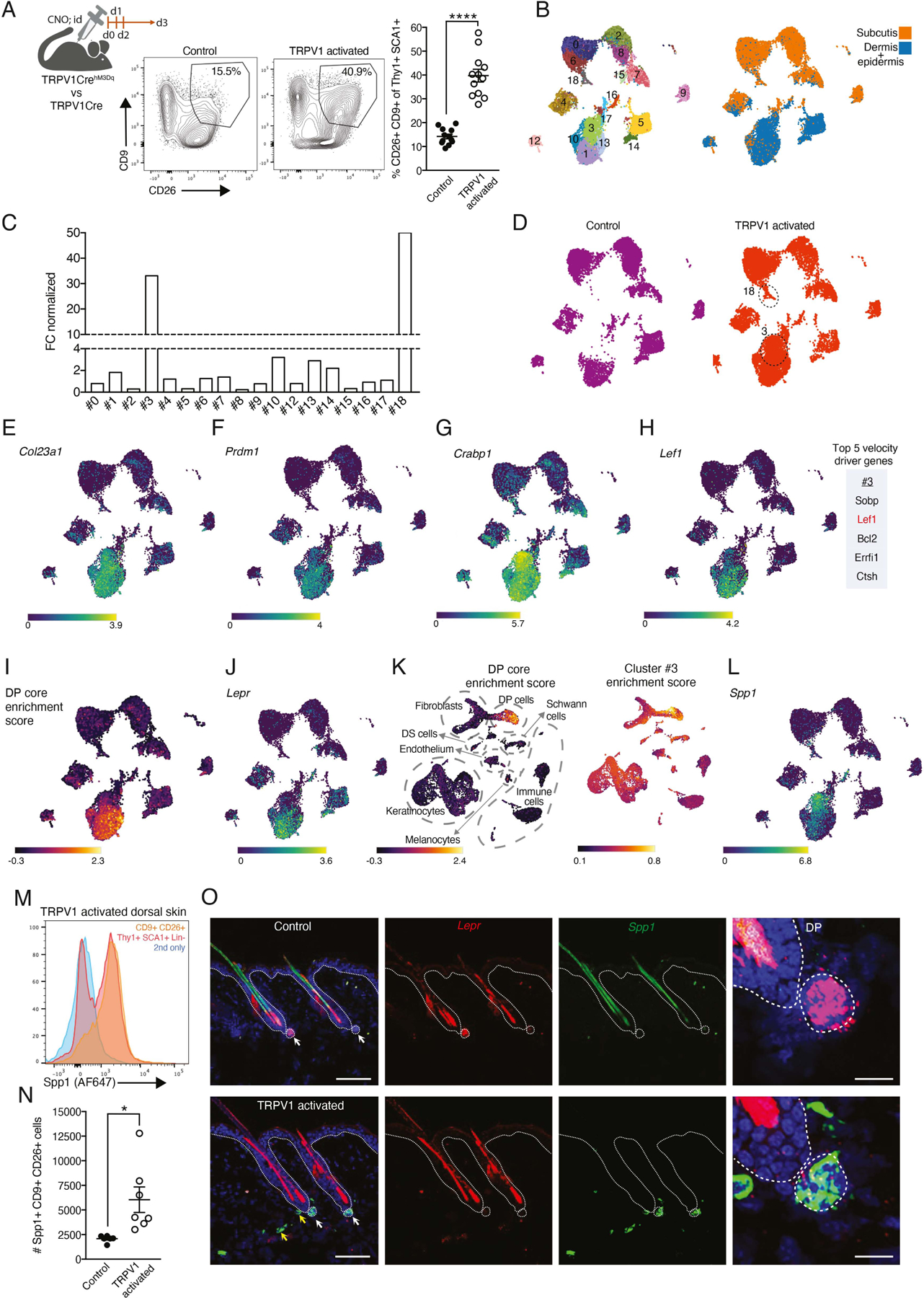
Repeated cutaneous TRPV1 activation triggered changes in dermal fibroblast gene expression. **(A)** Diagram of experimental design, representative FACS plots and quantification of CD9+CD26+ dermal fibroblasts percentage from Lin- Thy1+SCA1+ dorsal skin cells after three daily CNO intradermal injections from TRPV1 activated mice and their controls (****p<0.0001). **(B)** UMAP projection demonstrating the clustering of sorted Thy1+SCA1+ fibroblasts based on scRNAseq data, and the overlay of the Hashtag signal differentiating the subcutis and dermal clusters. **(C)** Normalized fold change (FC) calculation comparing cell numbers in dermal clusters between TRPV1 activated mice and their controls. **(D)** UMAP projection of sorted fibroblast scRNAseq data demonstrating cell distribution between clusters in the TRPV1 activated sample and control. **(E-H)** UMAP projection of sorted fibroblasts demonstrating the expression of **(E)** *Col23A1* **(F)** *Prdm1* **(G)** *Crabp1* and **(H)** *Lef1* as well as the list of top five velocity driver genes for cluster #3. **(I)** UMAP projection demonstrating calculated DP-core enrichment score and **(J)** *Lepr* expression. **(K)** UMAP projection of scRNASeq from Li et al.^41^ demonstrating its calculated DP-score (left) and cluster #3 enrichment score (right). **(L)** UMAP projection of sorted fibroblasts demonstrating the expression of *Spp1.* **(M)** Representative FACS histograms of anti-Spp1 staining of Lin- Thy1+SCA-1+ dermal cells (red) or CD9+CD26+ subset (orange) from the back skin of TRPV1 activated mouse. Secondary only staining control in blue. **(N)** Quantification of Spp1+CD9+CD26+ dermal fibroblasts from TRPV1 activated mice and controls (*p=0.017). **(O)** Representative images of RNAscope staining for *Spp1* (green) and the *Lepr* (red) on dorsal skin collected from TRPV1 activated mice and controls. Scale bar, 100um. White arrows point out DP; yellow arrows point out Spp1 expression out of DP. DP images scale bar, 25um.

Next, we analyzed the effects of repeated cutaneous TRPV1 activation on dermal fibroblasts. We found that three daily TRPV1 activations treatments with intradermal CNO injection induced significant changes in stromal cell composition identified in flow cytometry (FACS) by the lack of non-fibroblast lineage markers (CD45, Epcam, and CD31) and the expression of Thy1, SCA-1, CD26, and CD9 (Figure 1A and S2A). Thy1 is a pan-fibroblast and mesenchymal stem cells marker^30^, and SCA1 expression is mostly associated with a pre-adipocyte subset of fibroblasts^31,32^. CD9 and CD26 have been previously used to define different fibroblast subsets^33–36^. We also performed single-cell RNA sequencing (scRNAseq) analysis by sorting lineage negative (CD45- Epcam- CD31-), Thy1+ and SCA1+ cells from dorsal dermis and subcutis of TRPV1 activated mice and controls. A total of 18 cell clusters were identified, and labeling with Hashtag antibodies enabled the differentiation between dermal clusters (#1,#3,#5,#10,#13,#12,#14,#16 and #17) and subcutis clusters (#0,#2,#4,#6,#7,#8,#9,#15 and #18) (Figure 1B). All clusters expressed pan-fibroblast markers such as *Pdgfra* and *Col3a1* (Figure S2B).

The most notable changes in cluster size after TRPV1 activation were identified in dermal cluster #3 and the subcutaneous cluster #18 (Figure 1C and D). Cluster #3 became the largest dermal cell cluster, while cluster #18 comprised only 1.7% of the subcutaneous cell fraction (Figure S2C). In line with the FACS analysis, scRNAseq data demonstrated the expression of CD9 and CD26 (encoded by *Dpp4*) by cluster #3 cells and its adjacent clusters (#1, #10, #13; Figure S2D).

Dermal papilla (DP) cells are specialized niche fibroblasts regulating HFSCs and hair growth. Interestingly, cluster #3 and its adjacent clusters expressed genes typical of DP cells, such as *Col23a1*, *Prdm1*, *Crabp1* and *Lef1*^32,36–40^ (Figure 1E–H). Moreover, RNA velocity analysis identified *Lef1* as one of the top 5 pseudo-time driver genes of cluster #3 (Figure 1H and S2E). To assess cluster #3 transcriptional similarity to bona fide DP cells, we utilized a core DP gene signature adopted from Liu *et al*.^41^ to generate an enrichment score for our data set. On comparison, clusters #1, #10 and #13 had the highest DP score, indicative of bona fide DP cell identity (Figure 1I and S2F), while cluster #3 had an intermediate score (Figure 1I and S2F). For instance, cluster #3 expressed lower levels of DP marker such as *Lepr*^21,41^, *Igfbp3*^42^ and *Bcl2*^43^ (Figure 1J and S2G). We also assessed transcriptional similarities between cluster #3 and other skin derived cells, by comparing the cluster #3 transcription profile to a published skin scRNAseq dataset^44^. The DP core signature and cluster #3 enrichment score were both highest in the same cell subset in the published dataset (Figure 1K). Collectively, these findings suggest that cluster #3 cells represent a DP-like state that does not fully match bona fide DP marker expression.

Additionally, the DP gene Spp1^45^, linked to wound healing^46,47^ and triggering hair growth by directly engaging epithelial HFSC^45^, was highly enriched in cluster #3 (Figure 1L). We identified Spp1 protein expression in CD9+ CD26+ fibroblasts after TRPV1 activation (Figure 1M,1N and S2H). RNAscope analysis of dorsal skin from TRPV1 activated mice, revealed an enrichment of *Spp1+* cells concentrated at the HF base, with some co-expressing the DP-associated gene *Lepr*, and rarer *Spp1+* cells expressing low to no *Lepr* were distributed outside this area (Figure 1O and S2I). The cells located at the HF base are in a position similar to that previously defined as the DP compartment^48^. Thus, these findings indicate that Spp1+ fibroblasts expand near HFSCs in response to TRPV1 activation both inside and around HFs.

### Activation of cutaneous TRPV1 innervations triggers HF growth via CGRP and Spp1.

**Figure 2:**
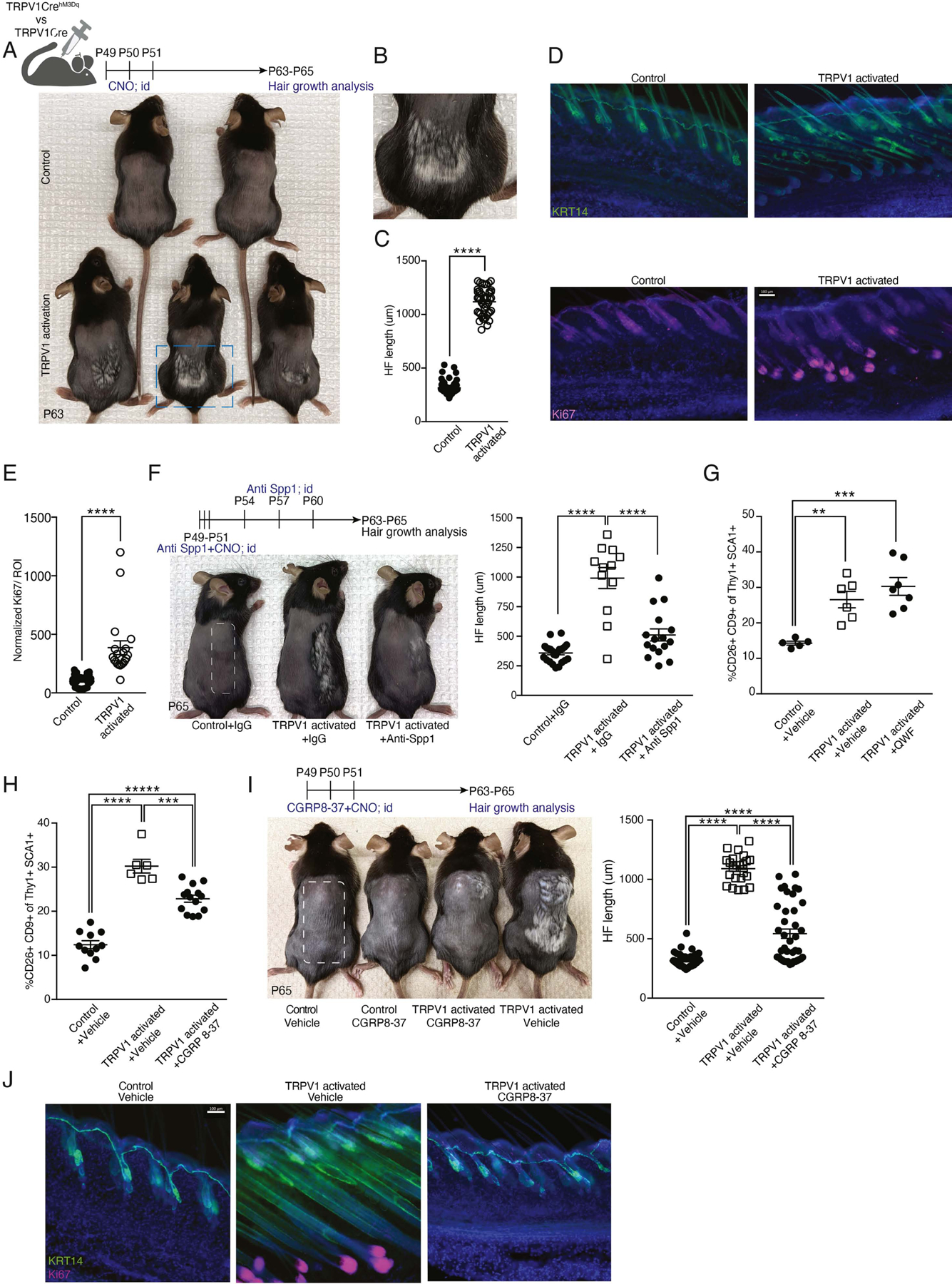
Repeated cutaneous TRPV1 activation triggered a CGRP-dependent acceleration of hair growth. **(A)** Diagram of experimental design and representative images from shaved TRPV1 activated mice and controls. Mice monitored for hair coat growth for 12 days after the last TRPV1 activation with CNO (postnatal day 63). **(B)** Enlarged dashed rectangle from panel A depicting hair growth in a TRPV1 activated mouse. **(C)** HF length quantification of dorsal skin from mice treated as in A. Data combined from 6 to 8 HF per mouse, taken from 6 mice per group (****p<0.0001). **(D)** Representative immunofluorescent images of dorsal skin from mice treated as in A. Staining with anti-keratin14 (KRT14; green) marking epidermal layer and Ki67 (purple) marking the HF bulb. DAPI stain in blue. Scale bar 100μm. **(E)** Quantification of normalized Ki67 MFI signal in the peri-follicular area of dorsal skin from mice treated as in A. Data combined from 4 regions of interest (ROI) per mouse taken from 5 TRPV1 activated mice and 9 controls (****p<0.0001). **(F)** Diagram of experimental design and representative images from TRPV1 activated mice and controls treated with CNO on half of their back skin (marked with dashed rectangle). Anti-Spp1 neutralizing antibody or IgG control were injected intradermally to the same skin area. Data are combined from 4 HFs per mouse collected from 6 controls, 3 TRPV1 activated + IgG treated and 4 TRPV1 activated + anti-Spp1 treated mice (****p<0.0001). **(G-H)** Percentage of CD9+CD26+ dermal fibroblasts from TRPV1 activated mice and their controls intradermally injected with **(G)** QWF (SubP antagonist) or vehicle (**p=0.005, ***p=0.0004), **(H)** CGRP8–37 (CGRP antagonist) or vehicle (***p=0.0001, ****p<0.0001). **(I)** Diagram of experimental design and representative images from TRPV1 activated mice and controls pre-treated intradermally with CGRP8–37 or vehicle and monitored for hair coat growth for 14 days. Quantification of HF length from 6 to 8 HFs per mouse, taken from 3–6 mice per group (****p<0.0001). **(J)** Representative immunofluorescent images of dorsal skin from mice treated as in I. Stained with anti-KRT14 (green), Ki67 (pink), and DAPI (blue). Scale bar, 100μm.

Given the impact of TRPV1 activation on dermal fibroblasts, we tested its effects on hair growth. Using DREADDs, we activated cutaneous TRPV1 innervations for three consecutive days in mice whose dorsal HFs were at telogen phase (period of hair growth-arrest; seven weeks old) and assessed the onset of new hair growth (i.e. anagen phase). After two weeks, TRPV1 activated mice had skin patches with anagen HFs while no hair growth was detected in controls (Figure 2A). TRPV1 activation also produced some amount of hair graying (Figure 2A, B), which is in line with a previous report demonstrating loss of melanocyte stem cells triggered by pain-induced stress^49^. Immunofluorescent staining confirmed HF morphology and the presence of Ki67+ early-stage hair matrix (Figure 2C, D and E). These findings are consistent with the early induction of DP-like fibroblasts and demonstrate a causal connection between TRPV1 activation and precocious hair growth.

To assess the engagement of the HF niche after TRPV1 activation, we adopted an approach used by Choi et al.^50^ and sorted HFSCs seven days after TRPV1 activation. The expression of seven key cell cycle and cytokinesis-related genes were analyzed (*Aurkb, Ccnb1, Ccnb2, Cdca2, Rad51, Prc1* and *Cdk1*) revealing a significant increase in their expression after TRPV1 activation (Figure S3A), indicative of proliferative activation of HFSCs. HFSC activation temporally followed the expansion of CD9+ CD26+ dermal fibroblasts, suggesting a role for these fibroblasts in mediating the precocious hair growth triggered by TRPV1 activation. Since Spp1 expression was enriched in CD9+CD26+ fibroblasts (Figure 1M and N), we tested its contribution to TRPV1-triggered hair growth. Treating TRPV1 activated mice with Spp1 blocking antibody significantly attenuated hair growth (Figure 2F), demonstrating a role for Spp1 in this context.

CGRP and SubP are the two main neuropeptides secreted by TRPV1 nociceptors^51,52^. We examined their role in CD9+ CD26+ fibroblast induction by pre-treating mice with their antagonists before TRPV1 activation. Unlike the SubP antagonist, QWF, which had no effect dermal fibroblasts (Figure 2G), the CGRP antagonist, CGRP8–37, significantly reduced both CD9+CD26+ fibroblast induction and hair growth, (Figure 2H, I and J), highlighting CGRP's involvement in TRPV1-induced fibroblast and hair growth phenotypes.

### Induction of CD9+CD26+ fibroblasts triggered by TRPV1 activation depends on macrophage CGRP sensitivity.

**Figure 3:**
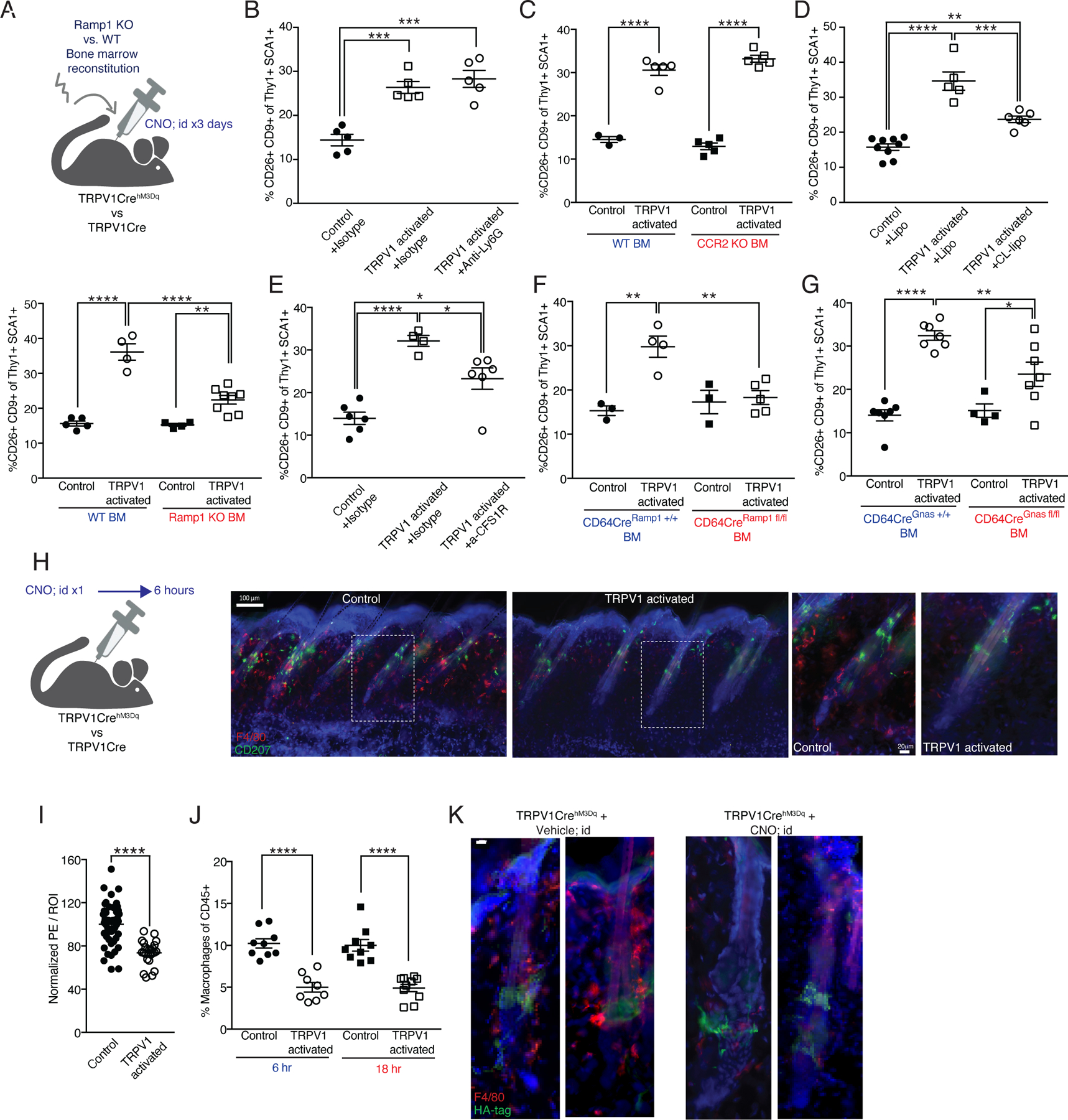
Macrophages rapidly respond to cutaneous TRPV1 activation and partake in mediating its effects on dermal fibroblasts. **(A-G)** Quantification of CD9+CD26+ dermal fibroblasts from TRPV1 activated mice and controls given three daily intradermal CNO injections. **(A)** (Upper) Diagram of experimental design. (Lower) Mice reconstituted with Ramp1 KO or wild type BM (**p=0.005,****p<0.0001). **(B)** Mice pretreated systemically with isotype control or anti-Ly6G antibody (***p=0.0005). **(C)** Mice reconstituted with CCR2 heterozygous or homozygous KO BM (****p<0.0001). **(D)** Mice pretreated systemically with clodronate (CL) or control liposomes (lipo) (**p=0.002,***p=0.0004,****p<0.0001). **(E)** Mice pretreated systemically with anti-CSF1R or isotype control (*p=0.029,****p<0.0001). **(F)** Mice reconstituted with CD64Cre^Ramp1+/+^ (control) or CD64Cre^Ramp1fl/fl^ BM (**p=0.003, 0.002). **(G)** Mice reconstituted with CD64Cre^Gnas+/+^ (control) or CD64Cre^Gnasfl/fl^ BM (*p=0.033,**p=0.007,****p<0.0001). **(H)** Diagram of experimental design and representative immunofluorescent images of dorsal skin from TRPV1 activated mice and controls after a single intradermal CNO injection. 150μm sections stained with anti-F4/80 (red), anti-CD207 (Langerin, green), and DAPI (blue). Scale bar 100μm. Dashed rectangles indicate enlarged area in images on the right 20μm scale bar. **(I)** Quantification of F4/80 normalized MFI signal in the peri-follicular area. Data combined from 8 region of interest (ROI) per mouse taken from 5 control and 3 TRPV1 activated mice treated as in H (****p<0.0001). **(J)** Percentage of dermal macrophages from TRPV1 activated and control mice at 6 and 18 hours after a single intradermal CNO injection (****p<0.0001). **(K)** Representative immunofluorescent images of HF from mice treated as in H. 50μm sections stained with anti-HA-tag (green), F4/80 (red), and DAPI (blue). Scale bar 10μm.

CGRP binds to a receptor-complex composed of calcitonin receptor-like receptor (*Calcrl*) and receptor activity-modifying protein-1 (*Ramp1*)^53^, expressed by various cell subsets including immune cells^54–57^. Our scRNAseq analysis revealed some expression of *Calcrl* but minimal *Ramp1* expression by cluster #3 fibroblasts (Figure S3B), suggesting indirect communication between TRPV1 neurons and these fibroblasts. We therefore tested whether hematopoietic CGRP-responsiveness partakes in mediating the effects of TRPV1 activation on dermal fibroblasts. We applied TRPV1 neuronal activation in chimeric mice reconstituted with bone marrow (BM) from mice knock out (KO) for Ramp1 or controls and detected a significant reduction in the induction of CD9+CD26+ fibroblasts in Ramp1 deficient chimeric mice (Figure 3A). This suggests that a hematopoietic cell subset responding to CGRP is involved in mediating CD9+CD26+ fibroblast induction. Since the time needed for hematopoietic reconstitution following whole-body irradiation causes mice to enter spontaneous late-onset hair growth, we could only measure fibroblast induction rather than precocious hair growth.

As previously mentioned, TRPV1 activation also led to neutrophil and monocyte infiltration into the skin. Thus, we tested these cells involvement in the induction of CD9+CD26+ fibroblasts. We depleted neutrophils with an anti-Ly6G antibody, which reduced their recruitment into the skin (Figure S3C) but did not inhibit CD9+CD26+ fibroblast induction after TRPV activation (Figure 3B). Similarly, CCR2-deficient BM reconstitution, which diminished Ly6C+ monocyte recruitment into the skin of TRPV1 activated mice (Figure S3D), had no impact on CD9+CD26+ fibroblast induction (Figure 3C). Of note, in the dermis of control mice, hematopoietic CCR2 deficiency did not significantly alter macrophage frequency eight weeks after reconstitution (Figure S3E). Taken together, these findings suggest that a hematopoietic-derived cell subset, but not newly recruited neutrophils or monocytes, is mediating the induction of CD9+CD26+ fibroblasts.

Next, we examined the role of dermal macrophages in the TRPV1-triggered fibroblast response. This was motivated by published work showing that most dermal macrophages post-irradiation are BM derived^58^, which we also validated (Figure S4A), and by evidence for macrophage-fibroblast functional interactions^59–61^. Depleting macrophages using clodronate liposomes or anti-CSF1R antibody significantly lowered CD9+CD26+ fibroblast percentage (Figure 3D, E, S4B). Additionally, we generated mice that have a macrophage-specific Ramp1 deficiency by crossing CD64-Cre mice with Ramp1-floxed mice (CD64Cre^Ramp1fl/fl^) and used these mice as BM doners for irradiated TRPV1^hM3Dq^ mice and controls. BM transfer from CD64Cre^Ramp1fl/fl^ mice reduced CD9+CD26+ fibroblast induction after TRPV1 activation (Figure 3F).

The CGRP receptor is a member of the G-protein-coupled receptors family (GPCR) signaling via the Gαs protein encoded by the *Gnas* gene^62^. Thus, to achieve conditional inactivation of CGRP signaling in macrophages, we genetically restricted the expression of *Gnas* specifically in macrophages by crossing *Gnas*-floxed mice with *CD64-Cre* mice (CD64Cre^Gnasfl/fl^). Irradiated TRPV1^hM3Dq^ mice and controls were reconstituted with *Gnas* deficient or control BM. Gnas-deficient BM transfer significantly reduced CD9+CD26+ fibroblast induction after TRPV1 activation (Figure 3G). Taken together, these finding demonstrate a role for macrophage CGRP sensitivity and Gαs signaling in mediating the effects of TRPV1 activation on dermal fibroblasts.

### TRPV1 neuronal activation reduces dermal macrophage frequency via CGRP.

Our finding that skin macrophages contribute to events downstream of TRPV1 activation, together with the evidence that macrophages express CGRP receptor components and functionally react to this neuropeptide^57,63,64^ led us to examine the effects of TRPV1 activation on dermal macrophages. Microscopy analysis 6 hours after a single round of TRPV1 activation detected a significant reduction in dermal and peri-follicular macrophage density (Figure 3H and I). FACS analysis confirmed this reduction in dermal macrophages both 6 and 18 hours after TRPV1 activation (Figure 3J and S4C). Spatial proximity between DREADD-expressing HF innervations and macrophages was confirmed by microscopy (Figure 3K). Six hours after TRPV1 activation, macrophage frequency was reduced in the innervated perifollicular region (Figure 3K). We also observed that in CCR2-deficient BM chimeric mice, repeated TRPV1 activation lowered dermal macrophage frequency compared to mice reconstituted with control BM (Figure S3E). This might occur due to lack for incoming monocytes (due to CCR2 deficiency) to replenish the loss of dermal macrophages triggered by TRPV1 activation.

**Figure 4:**
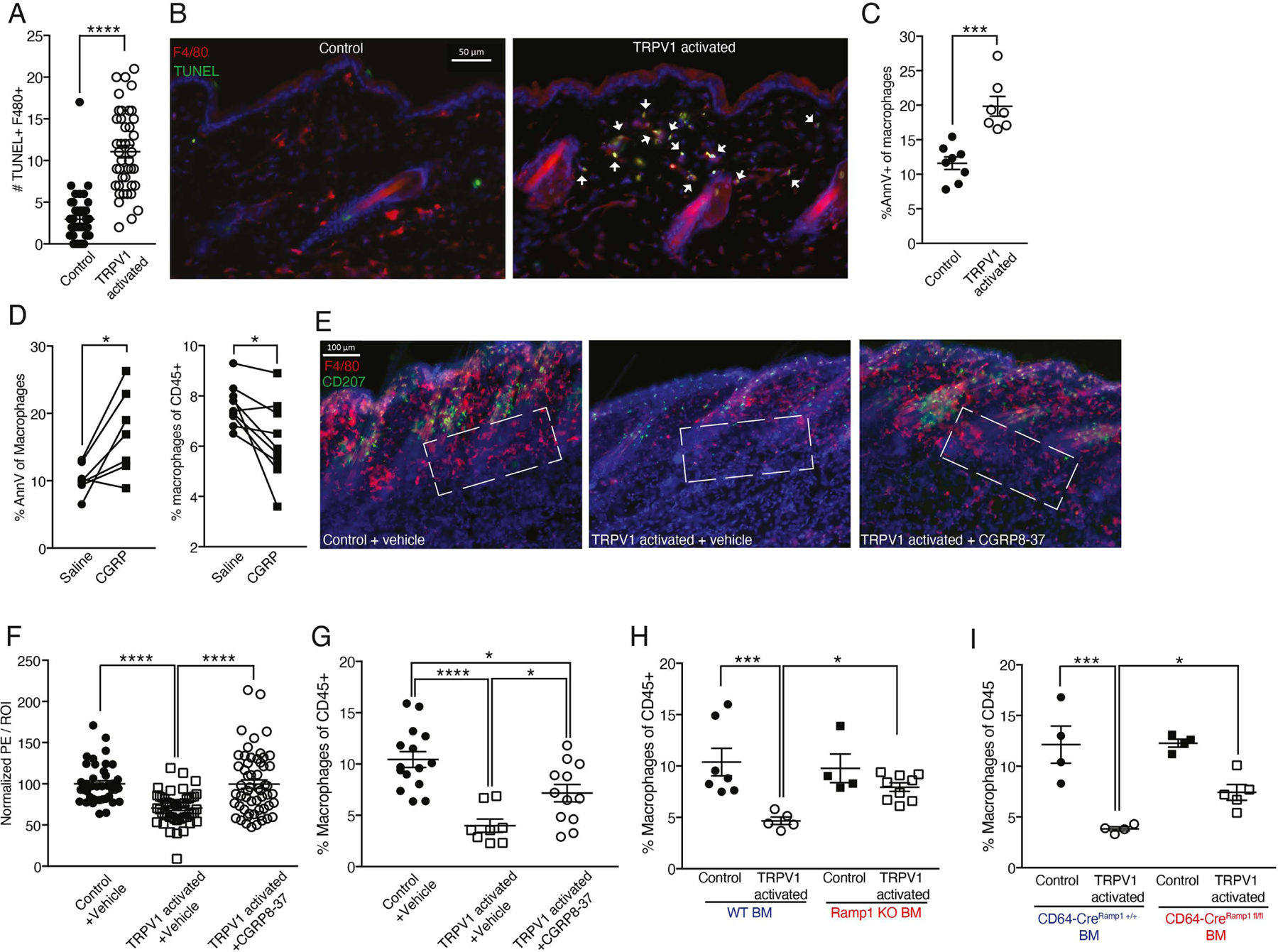
Dermal macrophage response to TRPV1 activation is CGRP dependent. **(A)** Quantification of TUNEL+ F4/80+ cell number per field of view from dorsal skin of TRPV1 activated mice and controls 3.5 hours after a single intradermal CNO treatment. Data are combined from 6 to 7 fields of view per mouse, taken from 7 mice per group (****p<0.0001). **(B)** Representative immunofluorescent images of 20μm skin sections from mice treated as in A. Sections stained with anti-F4/80 (red), TUNEL (green) and DAPI (blue). White arrowheads indicate F4/80+ TUNEL+ cells. Scale bar 50μm. **(C)** Percentage of Annexin V+ dermal macrophages from dorsal skin of TRPV1 activated mice and controls 45 minutes after a single CNO intradermal injection (***p=0.0002). **(D)** Mice back skin intradermally injected with CGRP on one side and vehicle on the other. (Left) Samples collected 45 minutes later and stained for annexin V as in C (*p-paired=0.017). (Right) samples collected 6 hours later and dermal macrophage frequency assessed (*p-paired=0.015). **(E)** Representative immunofluorescent images of 150μm dorsal skin sections from TRPV1 activated mice and controls pretreated with CGRP8–37 or vehicle followed by single intradermal CNO injection. Samples collected 6 hours later and stained with anti-F4/80 (red), CD207 (green), and DAPI (blue). Dashed rectangles indicate the lower follicle and peri-follicular area. Scale bar 100μm. **(F)** Quantification of F4/80 normalized MFI signal in the peri-follicular area from mice treated as in E. Data are combined from 6–8 ROIs per mouse taken from 5–8 mice per group (****p<0.0001). **(G)** Percentage of dermal macrophages from TRPV1 activated mice and controls treated as in E (*p=0.048,0.012,****p<0.0001). **(H)** Dermal macrophages frequency in dorsal skin of TRPV1 activated mice and controls reconstituted with Ramp1 KO or wild type BM. Chimeric mice given a single CNO injection and data collected 6 hours later (*p=0.04,***p=0.0009). **(I)** Dermal macrophages frequency in dorsal skin of TRPV1 activated mice and controls reconstituted with CD64Cre^Ramp1+/+^ or CD64Cre^Ramp1fl/fl^ BM. Mice treated with CNO as in H (*p=0.043,***p=0.0001).

To evaluate whether TRPV1 activation triggered macrophage apoptosis, we used TUNEL staining in sections and compared the frequency of TUNEL+ F4/80+ cells between TRPV1 activated mice and controls. We detected a 3.7-fold increase in the number of TUNEL+ F4/80+ cells in the dermis as early as 3.5 hours after TRPV1 activation (Figure 4A and 4B), indicating that dermal macrophages undergo neuron-induced apoptosis. Accordingly, Annexin V staining at 45 minutes after TRPV1 activation revealed a significant increase in macrophage surface phosphatidylserine content (Figure 4C), associated with early apoptosis.

Intradermal CGRP injection was able to phenocopy the increased Annexin V signal and reduced dermal macrophage frequency induced by TRPV1 activation (Figure 4D). To further explore the role of CGRP in mediating TRPV1 activation triggered effects on dermal macrophages, we pre-treated mice with the CGRP blocker, CGRP8–37, prior to activating TRPV1 cutaneous innervations. Microscopy and FACS analysis show that dermal macrophage frequency reduction was partially prevented by CGRP antagonist (Figure 4E–G). Specifically, CGRP8–37 protected from peri-follicular macrophage loss after TRPV1 neuron activation (Figure 4E). We also detected a reduction in type-2 (CD11b+) classical dendritic cell (cDC2) frequency, while type-1 classical dendritic cells (CD103+, cDC1) remained unchanged. However, the reduction in cDC2 frequency was CGRP independent, as pretreatment with CGRP8–37 had no effect on their frequency (Figure S4D). This observation is in accord with recent findings that highlight a role for TRPV1 innervation triggered and SubP mediated migration of dermal cDC2 via lymphatics^65^.

Next, we tested the possibility of CGRP acting directly on dermal macrophages to regulate their frequency. We hypothesized that transferring *Ramp1* KO BM should effectively maintain macrophage percentages following TRPV1 activation. Indeed, FACS analysis revealed that after TRPV1 activation, *Ramp1* KO chimeric mice had higher frequencies of dermal macrophage compared to mice reconstituted with control BM (Figure 4H). Furthermore, transferring BM with a macrophage-selective *Ramp1* deficiency (CD64Cre^Ramp1fl/fl^) was also effective in maintaining dermal macrophages frequency (Figure 4I). This demonstrates that macrophage CGRP sensitivity mediates their response to TRPV1 activation, suggesting a causal and direct connection between TRPV1 activation, CGRP and dermal macrophage fate. Moreover, these data are in line with prior evidence of a connection between low numbers of peri-follicular dermal macrophages due to apoptosis and onset of HF growth^66^.

### Dermal macrophage gene expression analysis implicates Axl in mediating effects of TRPV1 activation on fibroblasts.

**Figure 5:**
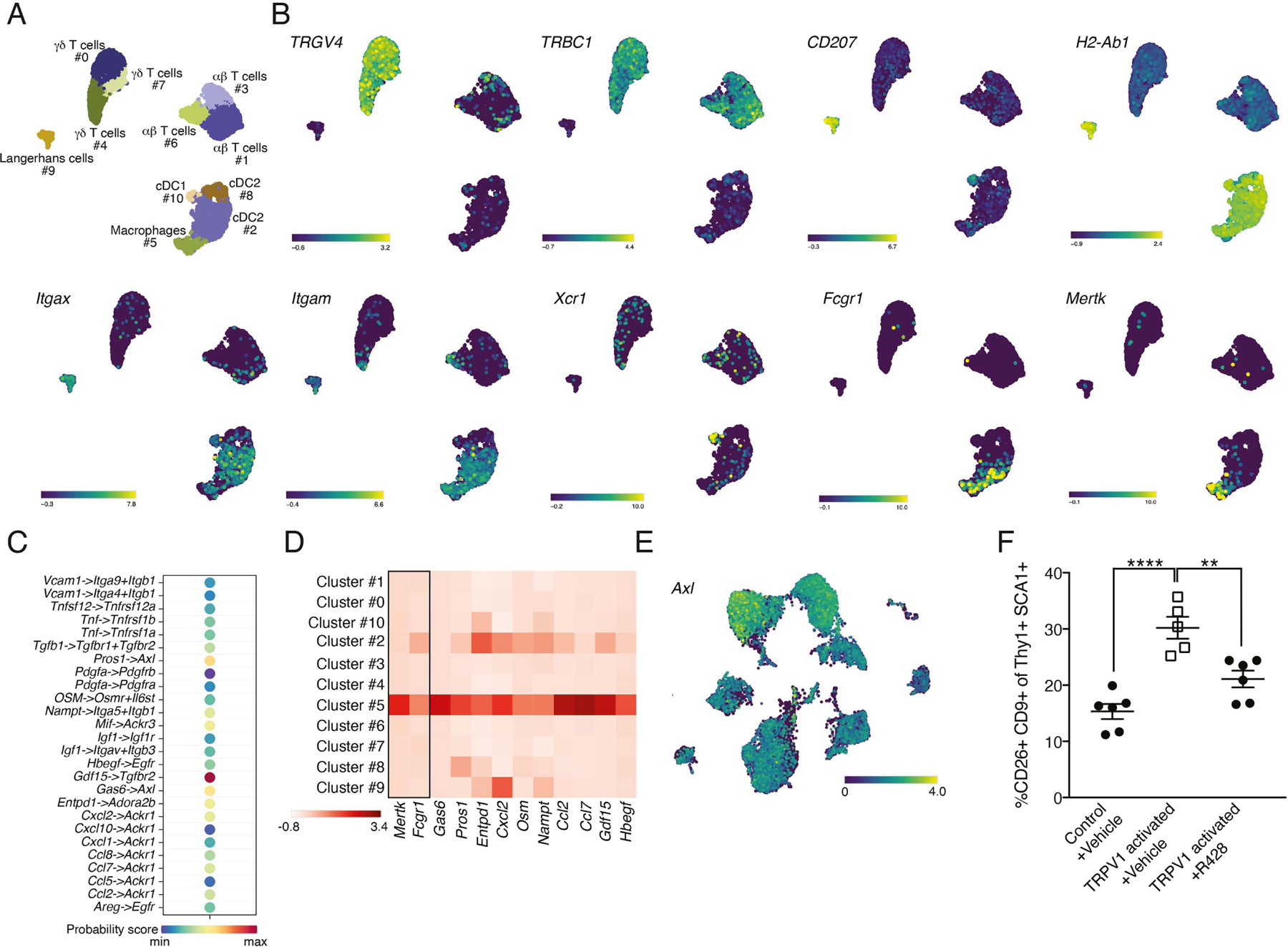
Dermal macrophage gene expression analysis and role for Axl signaling in effects of TRPV1 neuronal activation on fibroblasts. **(A)** UMAP projection demonstrating the clustering of sorted dermal CD45+ cells based of scRNAseq data. **(B)** UMAP projection demonstrating the expression of *TRGV4, TRBC1, CD207, H2-Ab2, Itgax, Itgam, Xcr1, Fcgr1* and *Mertk*. **(C)** Visualization of CellChat data demonstrating potential interactions and their probability between cluster #5 of dermal macrophages and cluster #3 of dermal fibroblasts. **(D)** Heat map visualization of gene expression for ligands identified by CellChat to have potential interactions with fibroblasts (x-axis) across all clusters (y-axis). Rectangle separates macrophage defining markers (*Mertk* and *Fcgr1*). Color encodes average gene expression. **(E)** UMAP projection of sorted fibroblasts demonstrating the expression of *Axl.* **(F)** Percentage of dermal CD26+CD9+ fibroblasts from TRPV1 activated and control mice pretreated with R428 or vehicle (**p=0.004,****p<0.0001).

To characterize the short-term effects of TRPV1 activation on immune cells, we performed scRNAseq analysis on sorted cutaneous CD45+ cells from naïve skin and 3.5 hours after TRPV1 activation with CNO. Our analysis identified cell clusters corresponding to γδ T cells (clusters #0, #7 and #4), αβ T cells (clusters #1, #3 and #6), Langerhans cells (cluster #9), cDC1 (cluster #10), cDC2 (clusters #2 and #8) and macrophages (cluster #5) (Figure 5A and B). At this early time point after TRPV1 neuronal activation no drastic changes in cluster size were detected (fold change ranging from 0.44 to 2.5; Figure S5A). Differential gene expression analysis combined with pathway analysis (using the online resource Enricher; https://maayanlab.cloud/Enrichr/) revealed that cluster #5 macrophages acquire a molecular signature associated with apoptosis-related genes, which was absent when preforming similar analysis for cluster #2 cells corresponding to cDC2 (Figure S5B).

Next, we investigated potential ligand-receptor interactions between cluster #5 macrophages from the CD45+ dataset (Figure 5A) and cluster #3 cells from the fibroblast dataset (Figure 1D) using CellChat analysis^67^. While revealing various potential molecular interactions between these clusters, Gas6/Pros1–Axl was among the ones with the highest probability score (Figure 5C). Further assessment of the expression profile of *Gas6* (growth-arrest-specific gene 6) and *Pros1* (vitamin K-dependent factors protein S) as well as other membrane-bound and secreted ligands, namely *Entdp1, Cxcl2, Osm, Nampt, Ccl2, Ccl7, Gdf15* and *Hbegf*, identified cluster #5 dermal macrophages to be their main source among immune cells (Figure 5D).

Since dermal macrophages undergo apoptosis shortly after TRPV1 activation (as indicated by TUNEL and Annexin V staining; Figure 4A–C), *Gas6* and *Pros1* seemed particularly relevant as these ligands bind phosphatidylserine that becomes exposed on the surface of apoptotic cells^68^. *Gas6* and *Pros1* are the activating ligands of TAM receptors (TYRO3, AXL and MERTK)^69,70^. Thus, TRPV1-triggered macrophage apoptosis may increase the availability of Gas6 and Pros1 in the dermis. Based on our fibroblast scRNAseq data, the TAM receptor *Axl* was expressed by both dermal and subcutis fibroblasts (Figure 5E), further supporting a potential Gas6/Pros1–Axl interaction. To test the contribution of Axl signaling to the induction of CD9+CD26+ fibroblasts, we pretreated TRPV1 activated mice and controls with the selective inhibitor of Axl kinase, R428^71^. R428 administration reduced the efficiency of CD9+CD26+ fibroblasts induction after TRPV1 activation (Figure 5F). Taken together, our findings show that dermal macrophages, which become apoptotic shortly after TRPV1 neuronal activation, express the Axl receptor ligands Pros1 and Gas6 that are displayed on apoptotic cell membranes^68^. While we cannot determine whether Axl is acting intrinsically in dermal fibroblasts, or indirectly via other Axl-expressing cells, our data highlights the Axl-signaling pathway as likely contributing to the TRPV1 triggered induction of CD9+CD26+ fibroblasts.

### Skin abrasion activates TRPV1 neurons and increases CD9+CD26+ fibroblast abundance in a TRPV1-dependent manner.

**Figure 6:**
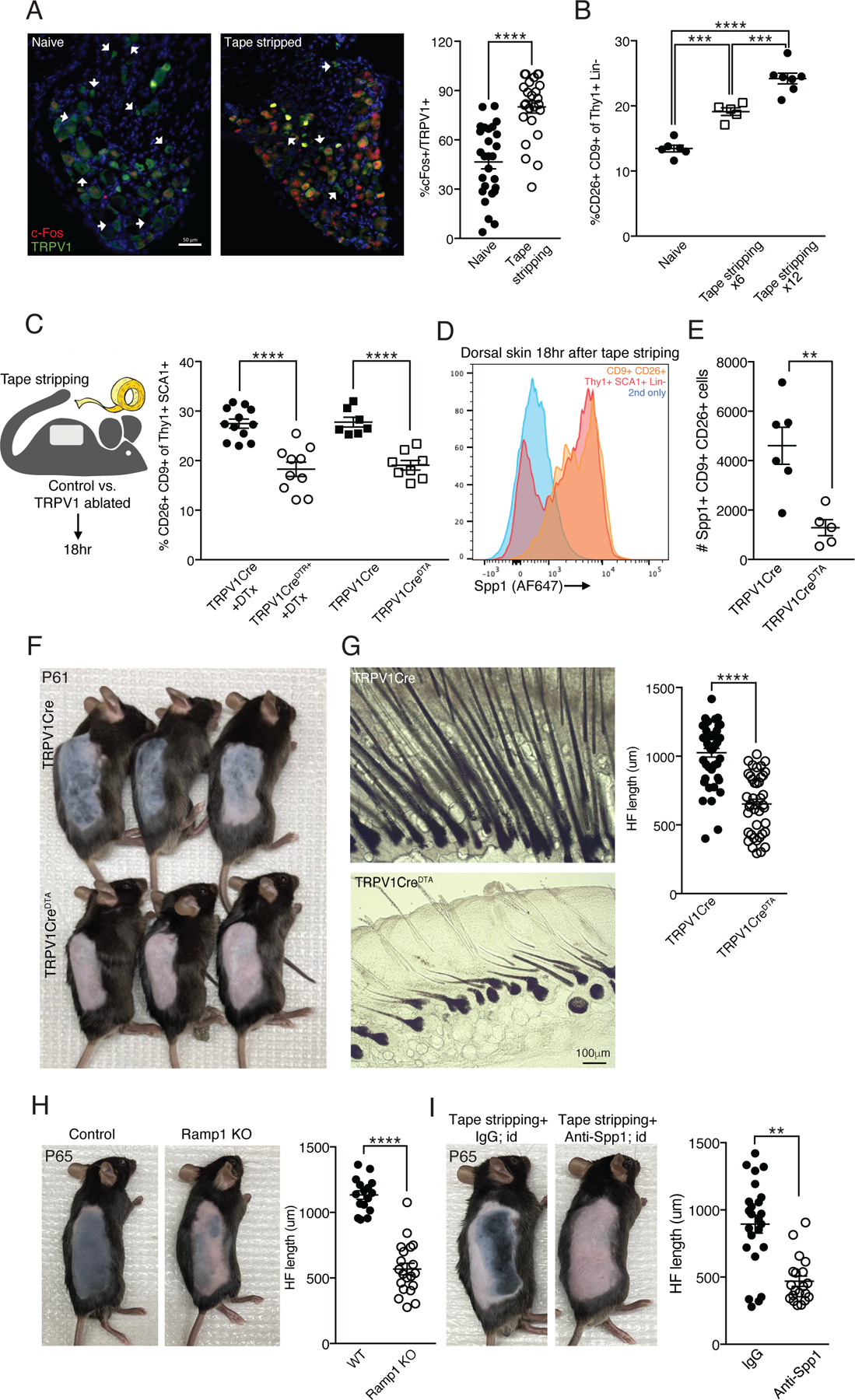
TRPV1 neuronal ablation affects dermal fibroblast reaction to tape stripping and attenuates post-wounding HF regeneration. **(A)** Representative immunofluorescent images and quantification of DRGs collected from mice with naïve back skin or 90 minutes after tape stripping. Stained with anti-c-Fos (red), anti-TRPV1 (green) and DAPI (blue). White arrowheads indicate neuron cell bodies that are TRPV1+ only. Data combined from 6–7 fields of view taken from 4 mice per group (****p<0.0001). Scale bar 50μm. **(B)** Percentage of CD9+CD26+ dermal fibroblasts from naïve back skin and 18 hours after tape stripping 6 or 12 times (***p=0.0004,****p<0.0001). **(C)** Diagram of experimental design and quantification of CD9+CD26+ dermal cell percentage 18 hours after tape stripping from the back skin of TRPV1Cre^DTR+^ treated with DTx or TRPV1Cre^DTA+^ mice and their controls (****p<0.0001). **(D)** Representative FACS histogram of anti-Spp1 staining. Dorsal skin treated as in C and gated on total Lin- Thy1+SCA-1+ cells (red) and the CD9+CD26+ cell subset (orange). Secondary only staining control in blue. **(E)** Quantification of Spp1+CD9+CD26+ dermal fibroblasts from TRPV1 ablated and control mice treated as in C (**p=0.005). **(F)** TRPV1 ablated mice and controls treated as in C and monitored for hair coat recovery for 12 days (postnatal day 61). **(G)** Representative bright field images of samples described in F. Data for HF length quantification collected from 8 HFs per mouse taken from 6 control and 5 TRPV1 ablated mice (****p<0.0001). **(H)** Ramp1 KO mice and controls treated as in F. HF length quantification from 4 HFs per mouse taken from 4 control and 5 Ramp1 KO mice (****p<0.0001). **(I)** Mice treated as in F and intradermally injected with anti-Spp1 or isotype control while being monitored for hair coat recovery for 16 days (postnatal day 65). HF length quantification from 4 HFs per mouse taken from 6 isotype control and 5 anti-Spp1 injected mice (**p=0.005).

Epidermal abrasions mostly compromise the epidermis, with only partial injury to the dermis in the case of second-degree abrasions^72–74^. Nevertheless, such epidermal abrasions can create an intense sensory experience and pain. To test whether epidermal abrasion in the form of repeated tape stripping can activate TRPV1 innervations, we extracted the dorsal root ganglia (DRG), which contains the soma of peripheral sensory innervations, from mice that had their back skin tape stripped and from controls with naïve skin. Staining DRGs for TRPV1 and the early neuronal activation marker c-Fos, we were able to compare the proportion of TRPV1+ c-Fos+ DRG neurons in the tape stripped and controls groups. This analysis showed a significantly higher proportion of TRPV1+ c-Fos+ neurons after tape stripping (Figure 6A), indicative of cutaneous TRPV1 neurons being activated.

Based on our findings using the DREADD system to activate cutaneous TRPV1 innervations, we hypothesized that in the context of epidermal abrasion, these innervations would mediate the post-wounding fibroblast reaction and hair re-growth. We first characterized the induction of CD9+CD26+ fibroblasts shortly after tape stripping and found an increased abundance of CD9+CD26+ fibroblasts as early as 18 hours after epidermal abrasion (Figure 6B). Moreover, the extent of tape stripping (i.e., number of repeated tape applications per skin area) affected the amount of CD9+CD26+ fibroblast induction in a ‘dose’ dependent manner, with 12 tape applications resulting in a higher percentage of cells compared to 6 tape applications (Figure 6B). To test the role of TRPV1 neurons in this response, we crossed *TRPV1-Cre* mice to ROSA-DTA or ROSA-iDTR mice, which enables lineage selective ablation of TRPV1 innervations (referred to as TRPV1CreDTA and TRPV1CreDTR, respectively). As expected, TRPV1 genetic ablation reduced heat sensitivity (measured by the hot plate assay; Figure S6A), without affecting the frequency of major dermal cell subsets such as keratocytes, Thy1+ fibroblasts and leukocytes (Figure S6B). Next, we assessed the effects of TRPV1 ablation at steady state and compared the percentage of CD9+CD26+ fibroblasts in naïve skin from TRPV1Cre^DTA+^ and control (TRPV1Cre) mice. We found a small but consistent reduction in CD9+CD26+ fibroblasts in naïve skin from TRPV1Cre^DTA+^ mice (22.2%; Figure S6C). Of note, dermal macrophage frequency in naïve skin or after tape stripping was unaffected by TRPV1 ablation (Figure S6D). Next, we tested the effects of TRPV1 ablation on the fibroblast response to tape stripping. We found that in diphtheria toxin (DTx)-treated TRPV1CreDTR mice and in untreated TRPV1Cre^DTA^ mice, the induction of CD9+CD26+ fibroblasts was reduced compared to controls (33.5% and 31.3% respectively; Fig 6C). This indicates that the induction of these fibroblasts after skin abrasion is TRPV1 dependent. Using FACS analysis, we found that Spp1 expression was enriched in CD9+CD26+ fibroblasts after tape stripping (Figure 6D, S6E). Moreover, there were less Spp1 expressing CD9+CD26+ fibroblasts in the tape stripped back skin of TRPV1 ablated mice compared to controls (Figure 6E). Combined with our data using the DREADD system for activating TRPV1 innervations in naïve skin, these observations highlight a central role for TRPV1 nociceptors in regulating the stromal cell compartment both at steady state and after wounding.

### TRPV1 ablation attenuates hair regrowth after skin abrasion.

Given that TRPV1 neuronal activation was sufficient to accelerate hair regrowth in naïve skin, we hypothesized that in the context of epidermal abrasion TRPV1 neuronal ablation would attenuate hair regrowth. Of note, the general coat condition of TRPV1 ablated mice was visually indistinguishable from controls. However, when we tape stripped dorsal skin of TRPV1Cre^DTA^, DTx-treated TRPV1Cre^DTR^ and control mice, hair regrowth was delayed in the TRPV1 ablated mice (Figure 6F, G and S6F, G). Thus, in the context of skin abrasion TRPV1 innervations are required for efficient HF activation and hair regrowth.

To exclude the possibility of any confounding TRPV1 expression on non-neuronal cells we crossed *Nav1.8-Cre* mice to conditional DTA mice (Nav1.8Cre^DTA^). *Nav1.8* expression is restricted to neuronal cell subsets^75^, and the use of the *Nav1.8-Cre* mouse line allowed efficient targeting of CGRP expressing neurons^76,77^. Of note, while there is a significant overlap between *TRPV1-Cre* genetic targeting and that of *Nav1.8-Cre* mice, *Nav1.8-Cre* recombination also targets some low threshold mechano-receptor (LTMR) neuronal subpopulations, which are not captured by the *TRPV1-Cre* line^76,77^. Nevertheless, Nav1.8 neuron ablation attenuated the rate of hair regrowth after tape stripping (Figure S6H and S6I), phenocopying the effects of TRPV1 ablation.

Our findings imply that CD9+CD26+ fibroblasts contribute to hair regrowth downstream of neuron activation. *Twist2-Cre* mice have been used to genetically manipulate papillary fibroblasts *in vivo*^78^. Our scRNAseq analysis confirmed that *Twist2* is significantly enriched among dermal fibroblast clusters, including cluster #3 (Figure S6J). To test whether *Twist2*-expressing fibroblasts are necessary for hair regrowth after tape stripping, we crossed *Twist2-Cre* mice with DTR expressing mice (Twist2Cre^DTR^) and used local DTx treatment (intradermal injection) to ablate fibroblasts in the dorsal skin. Local DTx treatment was sufficient to reduce the percentage of dermal Lin- (CD45- CD31- Epcam-) Thy1+ cells by half (Figure S6K), without any noticeable side effects and leaving the treated mice looking healthy. Assessing hair regrowth after tape stripping in DTx treated Twist2Cre^DTR^ mice and controls, we found that Twist2+ fibroblast-depleted mice phenocopied the attenuated hair regrowth displayed by TRPV1 ablated mice (Figure S6L). These data demonstrate the necessity of dermal fibroblasts for hair regrowth after epidermal abrasion and are consistent with dermal fibroblasts functioning downstream of sensory neurons to promote hair growth.

Mechanistically, our experiments using the DREADD system identified CGRP and Spp1 as factors necessary for accelerating hair growth after local TRPV1 activation in naïve skin. We therefore tested the role of these factors in supporting hair growth following skin abrasion. Ramp1 KO mice phenocopied the attenuated hair growth and reduced CD9+CD26+ fibroblast frequency displayed by TRPV1 ablated mice (Figure 6H and S6M). Also, repeated intradermal injection of Spp1 blocking antibody after tape stripping significantly reduced the rate of hair growth (Figure 6I). Finally, we tape stripped Spp1 KO and control mice, and monitored hair regrowth. In accord with the antibody blocking data, hair growth was attenuated in the Spp1 KO mice (Figure S6N). Collectively, these findings identify a role for CGRP and Spp1 in the process of post-epidermal abrasion hair regrowth.

## Discussion

Beyond the role of dermal TRPV1 innervation in thermal sensation and pain perception, these neurons also offer bidirectional feedback to the skin via local secretion of factors such as CGRP and SubP^79,80^. The present work examined the contribution of TRPV1 innervations to the process of hair regrowth. Skin in most mammals, including mice, features a dense array of HFs, that periodically grow new hair that collectively form fur coat. The latter plays a thermo-insulating role, but also protects skin from mechanical, chemical, and solar radiation insults. A significant skin injury is necessarily accompanied by loss of surrounding hair and this, in turn, necessitates new hair growth to restore the integrity of the fur coat. However, the exact mechanism of hair regrowth following skin injury remains poorly understood. Our data indicate that pain sensing innervations are important for promoting hair regrowth following skin injury.

We characterized the changes in dermal fibroblasts after repeated local activation of TRPV1 innervations in naïve skin using scRNAseq. This analysis revealed the expansion of a dermal CD9+CD26+ fibroblast population bearing a gene expression profile resembling that of DP cells, which are key for promoting the new hair growth cycle^37,38^. Further work will be required to determine the precise source of the CD9+CD26+ fibroblasts. Importantly, *Spp1* was among the top genes enriched in the TRPV1-induced fibroblast cluster (cluster #3). Spp1 is sufficient to promote the new hair growth cycle^45^, and our experiments using antibody blocking and genetic deficiency establish a role for Spp1 in induction of hair growth following skin abrasion and after TRPV1 activation. While our findings provide one likely mechanism by which CD9+CD26+ fibroblasts trigger new hair growth, they do not exclude additional contributions by these cells or the possibility of macrophages directly affecting HFSCs to augment hair growth.

The phenotypes induced by TRPV1 activation in naïve skin, namely (i) dermal macrophage apoptosis (ii) increased frequency of CD9+CD26+ dermal fibroblasts, and (iii) acceleration of hair regrowth, were all, at least partially, mediated by CGRP. Future work will define the mechanism by which macrophage apoptosis is triggered by TRPV1 activation. Our data also suggests that apoptotic macrophages communicate with dermal fibroblasts in a pathway that involves phosphatidylserine bound Gas6 and Pros1 engaging Axl signaling. Genetic studies with cell-type specific Axl-deletion will be needed to further explore this pathway.

Capsaicin is an irritant small molecule that induces pain by directly activating the TRPV1 receptor. Dermal injection of capsaicin and isoflavone for four weeks promoted hair growth associated with IGF-I expression in dermal papilla^81^. Our scRNAseq data of dermal fibroblasts did not identify changes in *Igf1* expression following TRPV1 activation with DREADDs. This may reflect differences in the method or duration of sensory neuron activation, but it seems likely that sensory neurons are equipped to promote hair growth by more than one pathway. This notion is also supported by the incomplete effect of deleting Ramp1 or blocking CGRP on TRPV1 activation-triggered phenotypes. Nevertheless, topical use of capsaicin in humans was reported to promote vellus hair growth^82,83^, which might suggest that our mechanistic findings may extend to humans.

Our experiments using the tape stripping model demonstrate that TRPV1 innervations, which are triggered by this type of wounding, can impact the skin’s reaction to such injury. Nevertheless, we did detect a reduction in CD9+CD26+ fibroblast frequency in naïve skin of TRPV1 ablated mice, perhaps indicative of homeostatic effects of sensory neurons on fibroblasts that are separate from those occurring following TRPV1 activation.

Pain is an integral part of injury and is experienced due to its underlying neuronal components that perceive tissue damage and noxious stimuli. This study reveals a causal connection between TRPV1 nociceptors and hair growth in naïve skin and after wounding. TRPV1 activation induced changes in stromal and immunological factors that are linked with supporting hair growth and dependent on the neuropeptide CGRP. Thus, the ability of pain and its underlying neuronal components to boost hair growth can be an advantageous evolutionary mechanism that connects the sensation of pain with improved skin healing and rapid restoration of the mechano- and thermo-protective fur coat.

## Limitations of the study

Further work is needed to define the mechanism of CGRP-triggered dermal macrophage apoptosis and to test the contribution of other neuron-derived signals to this process. The involvement of Gas6/Pros1-Axl communication in dermal macrophage-fibroblast interaction in the context of TRPV1 activation requires further testing using genetic approaches, and it remains likely that additional pathways are involved in promoting CD9+CD26+ fibroblast accumulation. Moreover, our work does not exclude the possibility of other TRPV1 triggered mechanisms contributing to the accelerated hair growth phenotype, for example, by directly impacting HFSCs. Finally, the use of the TRPV1Cre driver for genetic studies supports a role for TRPV1+ nociceptors in the pathway we describe but does not exclude a contribution by nociceptors of other types that express TRPV1 transiently during development.

## STAR Methods

### Resource Availability

#### Lead Contact

Further information and requests for reagents will be fulfilled by Dr. Jason Cyster (jason.cyster@ucsf.edu).

#### Materials Availability

No reagents generated in this study.

#### Data and Code Availability

- Single-cell RNA-seq data sets have been uploaded for deposit at GEO and are publicly available. Accession numbers are listed in the Key Resource table (see STAR Methods).
- This paper does not report original code.
- All other data reported in this paper will be shared by the lead contact upon request.

### Experimental model and study participant details

#### Mice

We generated TRPV1hM3Dq mice by crossing homozygous TRPV1-Cre mice^84^ (B6.129-Trpv1tm1(cre)Bbm/J; provided by Allan Basbaum, University of California in San Francisco) with heterozygous chemogenetic floxed hM3Dq mice^85^ (B6N;129-Tg(CAG-CHRM3*,-mCitrine)1Ute/J; Jackson laboratories). iDTR mice^86^ (C57BL/6-Gt(ROSA)26Sortm1(HBEGF)Awai/J), DTA mice^87^ (B6.129P2-Gt(ROSA)26Sortm1(DTA)Lky/J) and Nav1.8-Cre mice^75^ (B6.129(Cg)-Scn10atm2(cre)Jwo/TjpJ) were from Jackson Laboratories. Twist2-Cre females^88^ (B6.129X1-Twist2tm1.1(cre)Dor/J; provided by Jeffery Bush, University of California in San Francisco) were crossed to iDTR males. Ramp1 KO mice^18^ (B6.129S2(Cg)-Ramp1tm1.1Tsuj/WkinJ) were purchased from Jackson Laboratories. CD64-Cre mice^89^ were provided by Russell Vance, UC Berkeley. Spp1 KO mice^46^ (B6.129S6(Cg)-Spp1tm1Blh/J) and their controls (C57BL/6J) were purchased from Jackson Laboratories. Gnasf/f mice^90^ were from an internal colony and had been backcrossed to C57BL/6J 10 times. C57BL/6J and BoyJ (CD45.1) mice were bred in an internal colony. Ramp1f/f mice (C57BL/ 6N-Ramp1<tm1c(EUCOMM)Wtsi>/H) were provided by Pierangelo Geppetti, University of Florence, Italy.

Breeding was done in an internal colony, housed under 12-hour light: 12-hour dark cycles and given ad libitum access to food and water. Either 7 or 12-week-old mice of both sexes were used for experiments. Littermate and cage mate controls were used for experiments and mice were allocated to control and experimental groups randomly. Specifically, TRPV1 activated mice were co-caged with their littermates controls (DREADD negative TRPV1-Cre positive). Sample sizes were chosen based on previous experience to obtain reproducible results and the investigators were not blinded. Animals were housed in a pathogen-free environment in the Laboratory Animal Resource Center at the University of California, San Francisco, and all experiments conformed to ethical principles and guidelines that were approved by the Institutional Animal Care and Use Committee.

#### DREADD activation

TRPV1hM3Dq expressing mice and their controls (TRPV1Cre) were anesthetized with isoflurane and an area of 2x2cm of dorsal hair was shortened with clippers. A total of 150μl of CNO (diluted 1.3μg/ml in PBS) was injected intradermally in the shaved patch of skin, with about 6 injection points of 25ul each which were evenly spaced in the shaved area. About 10 minutes upon awakening from anesthesia, mice showed mild pain related behaviors like a slightly hunched posture and reduced movement, which faded after approximately three hours. This procedure was repeated for three consecutive days in the fibroblast assessment experiments, with mice being sacrificed on the fourth day. Throughout the course of the CNO treatment the mice remained healthy in appearance and maintained their weight. For studies assessing dermal macrophages, mice were euthanized 6 hours after a single CNO injection.

### Methods details

#### Hot Plate test

Mice (TRPV1hM3Dq mice, TRPV1Cre^DTA^ mice and either DREADD-negative or DTA-negative controls) were brought into the testing room 30–60 minutes before the procedure for acclimation. The surface of the hot plate was cleaned and heated to a maximum temperature of 53°C. TRPV1hM3Dq mice and their controls were lightly anesthetized and receive an intraplanar injection of 40 μl CNO (2.6μg/ml dilution in PBS) into both hind paws. Once fully awakened, which took about five to ten minutes, a mouse was placed on the hot plate and the latency to show a nocifensive response such as hind paw withdrawal, licking, stamping, leaning, or jumping is recorded. The mouse was immediately removed once this response was observed. If there was no response within about 40 seconds, the test was terminated, and the mouse removed from the hotplate to prevent heat-related injury. The surface of the hotplate was cleaned with 70% ethanol between the testing of each mouse.

#### Immunohistochemistry

Dorsal skin samples from TRPV1 activated mice and controls treated with CNO intradermally for three consecutive days were euthanized and skin patches of 2cmx2cm were collected. Samples were fixated with 10% Neutral Buffered Formalin for a maximum of 24 hours and transferred to the UCSF Histology and Biomarker Core for further processing, sectioning and hematoxylin and eosin staining. Back skin samples form naïve control (TRPV1Cre) mice or after repeated CNO treatment were prepared in a similar manner and trichrome stained in the same core facility.

#### Flow cytometry

Single cell suspensions of dorsal skin were prepared at the indicated time points of CNO treatment. Fibroblast and macrophage analysis with flow cytometry or cell sorting was based on the protocol of Boothby et al.^33^. A skin patch of 2cmx2cm was dissected and the epidermal/dermal fraction separated from the subdermal fraction by tautly pinning the skin and roughly scraping off the subcutis with forceps until no dermal adipose tissue remained. Then, the epidermal/dermal fraction was minced finely with scissors, re-suspended in 1 ml of digestion mix (composed of 0.25mg/ml Liberase, 0.5mg/ml hyaluronidase and 0.1mg/ml DNase in RPMI with 1mM Hepes, and 2% fetal bovine serum), and incubated in a heated shaker at 37°C at 1000 rpm for 90 minutes. An additional 1ml of RPMI/Hepes/FBS media containing 1mM EDTA was added and the suspension, vortexed and then strained into a 50ml conical tube through a 100um strainer. Another 5ml of media was added to thoroughly wash the cell strainer and the suspension was centrifuged, re-suspended in staining buffer (PBS containing 1% NBCS, 0.5 mM EDTA and 0.05% Sodium Azide) for cell counting and staining. For intracellular anti-cytokeratin 14 and Spp1 staining cells were fixed and permeabilized using the Cytofix/Cytoperm kit Samples were analyzed on BD LSR II or BD FACSymphony flow cytometers. Data were analyzed using FlowJo software (v.10.6.2).

#### Single cell RNA sequencing of dermal fibroblasts and analysis

Back skin from DREADD-expressing and control mice (three mice per group) treated daily with a CNO intradermal injection for three consecutive days, were dissected, separated into the subcutis and dermal-epidermal fractions and then digested. The dermal-epidermal and subcutis fractions were stained separately with TotalSeq^™^ anti-mouse Hashtag antibodies to mark the cells’ anatomical location. Thy1^+^ SCA1^+^ stromal cells (CD45^−^CD31^−^ EpCAM^−^) were sorted based on the gating strategy depicted in Extended Data Fig. 2a, with the exception of cytokeratin 14 as it involves intercellular staining. The dermal-epidermal and subcutis fractions from TRPV1 activated and control samples were pooled together and sorted using a BD FACSAriaII with a 100-μm nozzle into RPMI medium supplemented with 10% FBS.

Treatment groups were run on separate lanes of a 10X Chromium chip with 3′ v.2 chemistry (10X Genomics) as per the manufacturer’s instructions by the UCSF Institute for Human Genetics Sequencing Core. Transcripts captured in all the cells encapsulated with a bead were uniquely barcoded using a combination of a 16 bp 10x Barcode and a 10 bp unique molecular identifier (UMI). cDNA libraries were generated using the Chromium^™^ Single Cell 3’ Library & Gel Bead Kit v3 (10x Genomics) following the detailed protocol provided by the manufacturer. Libraries were sequenced with the NovaSeq 6000 platform (S1 Cartridge, Illumina) in 150 bp paired-end mode. The hashtag library was demultiplexed using CellRanger software (version 7.0.0)^91^, discriminating between spliced and unspliced transcripts. Aligned spliced reads were used to quantify the expression level of mouse genes and generation of gene-barcode matrix. Subsequent data analysis was performed using Seurat R package (4.0.4)^92^ and Scanpy (1.8.2)^93^. Quality control was performed, and viable cells were selected by excluding cells with features lower than 200 and above 4000, as well as cells having more than 5% of mitochondrial transcripts. Dermal and epidermal derived cells were demultiplexed with the HTODemux function integrated in Seurat with standard settings and dermal derived cells were included in the analysis all doublets were removed for the analysis. 2000 most variable genes used for the anchoring process were used for downstream analysis to calculate principal components, after log-normalization and scaling. Principle component analysis (PCA) was used for dimensionality reduction and to visualize a uniform manifold approximation and projection (UMAP) of the identified clusters. P-values comparing gene expression of clusters and samples were calculated with the FindMarker function in Seurat. We identified one cluster contaminated with keratinocytes (cluster #11) and three clusters contaminated with immune cells (clusters #19-#21) which we have excluded from our analysis. Gene list was generated using an adjusted p-value cutoff <0.05 and expression in the indicated cluster cutoff >50%. Marker genes for each cluster were identified by using FindAllMarkers function in Seurat. The spliced reads and unspliced reads were counted by the Velocyto (0.17). Velocity driver gene for each cluster were identified following the scVelo (0.1.25) pipeline. Gene signature scores were calculated using the AddModuleScore function in Seurat. DP score signature was obtained from Liu Y. et al^41^ and cluster 3 signature genes were calculated with the FindMarker function in Seurat. Publicly available data sets were downloaded and reanalyzed^44^ using the Seurat pipeline. Cluster annotation for dataset from Liu Y. et al^41^ was done manually identifying keratinocytes (KRT14+ Epcam+) fibroblasts (Col1a1+ Pdgfra+) DP-cells (Crabp1+ Lef1+) endothelium (Pecam1+ Lyve1+) immune cells (Ptprc+ Epcam-) DS-cells (Acta2+ Mylk+) melanocytes (Tyrp1+ Sox10+) and Schwann cell (Mpz+ Sox10+).

#### Single cell RNA sequencing of dermal immune cells and analysis

Back skin from TRPV1 activated and control mice (two mice per group) were collected 3.5 hours after a single intradermal CNO injection. The subcutis was removed mechanically and the dermal-epidermal fractions were then digested. TotalSeq^™^ anti-mouse Hashtag antibodies were used to mark the different mice before pooling all control and TRPV1 activated samples together for staining and sorting. CD45+ were sorted using a BD FACSAriaII with a 100-μm nozzle into RPMI medium supplemented with 10% FBS. Sequencing, quality control, viable cells selection and cluster analysis were performed as described for the fibroblasts scRNAseq dataset. Clusters contaminated with non-immune cells were excluded from the analysis. Cell-cell interactions were analyzed using the CellChat R package (1.6.1) with standard parameters. For pathway analysis, the cluster defining genes of #C5 and #C2 macrophages were identified with the FindMarker function in Seurat and were analyzed using the Enrichr tool^94,95^. The pathway enrichment was calculated using standard Enrichr settings.

##### Skin immunofluorescent staining and image quantification.

Sample preparation, section and staining are based on methods described by Salz and Driskell^96^. Briefly, full thickness skin samples were collected and fixed in 4% Paraformaldehyde, before being frozen in OCT compound in cryo-block and kept in −80°C. Samples were sectioned with a cryostat at 150 μm thickness and transferred to PBS using forceps. Sections were then stained in 1.5ml eppendorfs with PE-conjugated anti-F4/80 (1:400) and APC-conjugated anti-CD207 (1:400) for macrophage analysis, or FITC-conjugated anti-cytokeratin14 (1:400) and APC-conjugated anti-Ki67 (1:200) for HF length analysis. Antibodies were diluted in staining buffer (PBS containing 10% mouse serum, 0.3% triton and 1% FBS) for 48 hours at 4°C under gentle rotation. Sections were transferred to a new tube for washes, and then mounted on glass cover slip with Fluoroshield^™^ with DAPI (Sigma-Aldrich) mounting medium under a dissection microscope to preserve orientation. Images were captured with a Zeiss AxioObserver Z1 inverted microscope with Apotome attachment. In quantification analysis each data point representing one HF. Skin sample staining for HA-tag, 50 μm thick sections were mounted onto Superfrost plus glass slides and stained with anti HA-tag (1:400) at 4°C for 48 hours in staining buffer. Slides were washed and stained with goat anti-rabbit secondary antibody for 18 hours.

##### RNAscope tissue staining and imaging.

DREADD expressing mice and their controls were given three daily TRPV1 neuronal activation sessions with CNO intradermally injected as described above. Skin samples were collected, quickly embedded in OCT and make 12 µm sections in cryostat at −20 °. Frozen sections were processed for RNA scope using RNAscope multiplex fluorescent detection kit v2 according to manufacturer’s protocol. The following probes were used: Spp1, Lepr. TSA Vivid Fluorophore Dyes 520 and 650 were used to detect target signal in different channel. Images were acquired on Olympus FV3000 confocal laser scanning microscope.

##### TUNEL staining and image quantification.

Skin samples were collected, fixed and frozen as described above, and then sectioned with a cryostat at 15–25 μm thickness, mounted onto Superfrost plus glass slides, and stained for TUNEL (In Situ Cell Death Detection Kit, Fluorescein, Roche) according to manufacturer instructions. TUNEL-stained sections were incubated overnight with PE-conjugated anti-F4/80 (1:400). Then, sections were washed in PBS, mounted with Fluoroshield^™^ with DAPI mounting medium and imaged on a Zeiss AxioObserver Z1 inverted microscope with Apotome attachment.

##### HF length quantification.

For the assessment of repeated DREADD-induced TRPV1 neuronal activation on HF growth, the entire back skin of 7-week-old cage mate and littermate TRPV1 activated mice and their controls were shaved with clippers. A total of 300ul of CNO (1.3μg/ml dilution in PBS) were intradermally injected at several sites evenly spread along the mouse back (25ul per injection site; a total of 12 injection sites per mouse) for three consecutive days. Mice were euthanized 14 days later, and hair growth was documented by standardized photographs. An approximately 1cm wide strip of midline back skin was dissected for immunofluorescent thick section staining and imaging.

#### Quantitative RT-PCR

For assessment of cytokine gene expression in TRPV1 activated or control mice after repeated CNO treatment, skin patches the size of 2cmx2cm were separated into epidermal/dermal fraction and subdermal fraction by tautly pinning the skin and roughly scraping off the subcutis with forceps. The epidermal/dermal fraction was finely chopped with scissors, and total RNA from both fractions was extracted using RNeasy kit and reverse-transcribed using M-MLV reverse transcriptase. qPCR was performed using Power SYBR Green with an Applied Biosystems StepOnePlus instrument. Data were analyzed with the comparative Ct (2−ΔΔCt) method, using HPRT as housekeeping gene control.

For assessment of cell-cycle and cytokinesis associated genes in HFSC, TRPV1- DRERADD expressing mice or controls were treated for three consecutive days with a daily intradermal CNO injection. Seven days after the last CNO treatment HFSC were sorted based on the gating strategy depicted in Extended Data Fig. 3a (based on a strategy described by Wang et al^97^ and Choi et al^50^). About 0.1×10^6^ sorted cells were snap frozen using liquid nitrogen.

#### In vivo neutrophil depletion, anti-Csf1r administration and intradermal anti-Spp1 injection

TRPV1 activated mice and their controls were intraperitoneally administered 200ug of anti-Ly6G 24 hours prior to starting a three-day CNO intradermal treatment. IgG2a isotype was used at the same dosage as control. Anti-Ly6G treatment on its own did not affect the percentage of CD9+ CD26+ fibroblasts. Depletion of Ly6G+ cells was confirmed by flow cytometry as depicted in Extended Data Fig. 3c.

Anti-Csf1r neutralizing antibody (500ug per mouse) was injected intraperitonially to TRPV1 activated mice and their controls only 48 and 24 hours prior to starting a three-day CNO intradermal treatment. Anti-Csf1r treatment on its own did not affect the percentage of CD9+ CD26+ fibroblasts. Depletion efficiency was assessed 18 hours after the last CNO treatment, which is day 5 after the last Anti-Csf1r administration, on dorsal skin samples of control mice (Extended Data Fig. 3e).

Anti-Spp1 neutralizing antibody was injected intradermally (150ug per mouse) to TRPV1 activated mice, starting at the first CNO treatment and every third day until mice were sacrificed and skin samples collected for hair growth analysis. Anti-Spp1 intradermal injection did not have any effects on control (DREADD negative) mice. The same dosing and injection protocol were applied for assessing Spp1 blocking effects on hair growth after tape stripping, with the first anti-Spp1intradermal injection administered 30 minutes after tape stripping.

#### CGRP8–37, QWF, and Axl inhibitor treatments

For the assessment of CGRP inhibition on fibroblasts, TRPV1hM3Dq expressing mice and their controls had a 2cmx2cm of dorsal skin patch was shaved with clippers. CGRP8–37 (0.2μg in 25ul PBS per injection site; a total 6 injection sites per skin patch) or PBS were intradermally injected one hours prior to every CNO treatment for three consecutive days. Mice were sacrificed and samples collected on the fourth day. For the assessment of CGRP inhibition on HF growth, the entire back skin of TRPV1activated mice and their controls was shaved with clippers. CGRP8–37 (0.2 μg in 25 μl PBS per injection site; a total of 12 injection sites per mouse) or PBS were intradermally injected one hour prior to every CNO treatment for three consecutive days. Mice were sacrificed and samples collected between the 12^th^ and 14^th^ day after the last CNO treatment. For the assessment of CGRP inhibition on macrophages, the shaved skin was injected with CGRP8–37 or PBS 18 hours prior to CNO injection. Another equal dosage of CGRP8–37 was mixed with CNO, which was injected intradermally (25ul per injection site; a total 6 injection sites per skin patch). Mice were euthanized 6 hour later.

For the assessment of substance P inhibition on fibroblasts, we used QWF which is an NK-1R antagonist as well as a blocker of the relevant human and mouse Mrgprs^98^. We injected the vehicle PBS or QWF intradermally into a 2cmx2cm patch of shaved dorsal skin one hour before every CNO treatment for three consecutive days (0.4μg in 25ul PBS per injection site; a total 6 injection sites per mouse). QWF and CGRP8–37 administration protocols were first tested on control (TRPV1Cre) mice and were found to have no effects on hair growth, dermal fibroblasts, or macrophages.

To inhibit AXL activity, R428 was used. 2mM concentration in ethanol was applied to TRPV1hM3Dq mice topically an hour before CNO was intradermally injected to the same back skin area. This was repeated for the three consecutive days. Samples collected for analysis on the fourth day.

#### Bone marrow chimera generation

To produce chimeras, 8-week-old TRPV1hM3Dq expressing mice and their controls were lethally irradiated with 1300 rad in split doses (separated by 3 hours) from a cesium source and reconstituted with 5 × 10^6^ bone marrow cells of indicated types by intravenous injection. Mice were analyzed 7–8 weeks later.

#### Clodronate liposomes treatment

Clodronate or control liposomes were intraperitoneally injected 2 days prior to starting a three-day CNO intradermal injection treatment. Depletion efficiency was assessed 18 hours after the last CNO treatment, which is day 5 after the liposome administration, on dorsal skin samples of control mice (Extended Data Fig. 3e). Clodronate liposome injection on its own did not affect CD9+ CD26+ fibroblasts percentages.

#### Diphtheria toxin treatment

For systemic ablation of TRPV1-lineage cells, TRPV1^DTR+^ mice were given an intraperitoneal injection with two doses of 10 ng/g body weight of DTx dissolved in PBS at 1ug/ml concentration. Injections were spaced every third day, with the first injection between seven and ten days before every experiment. DTx administration continued until mice were euthanized. Control mice were DTR negative and were given the same injection regimen and dosage as TRPV1^DTR+^ mice.

For local ablation of Twist2-lineage cells, Twist2^DTR+^ mice had a patch of dorsal skin covering about half the back right of the midline shaved with clippers and intradermally injected with 100ul of DTx dissolved in PBS at 0.1ug/ml concentration (25ul per injection point, four injection points per mouse). Injections were given at 48, 24 and 2 hours before tape stripping, as well as 24 and 48 hours after wounding.

#### Tape stripping and HF regrowth quantification

For the assessment of HF regrowth after epidermal abrasion, 7- to 8-week-old TRPV1^DTA+^, Nav1.8^DTA+^, TRPV1^DTR+^ (pretreated systemically with DTx) or Twist2^DTR+^ mice (pretreated with DTx intradermally) and their controls were anesthetized with isoflurane. Once fully anesthetized, a dorsal skin patch covering about half the back right of the midline was first shortened with clippers before applying depilatory cream (Nair) for 30 seconds and then gently wiping clean with wet paper towels. Depilated skin was allowed to dry for an hour to four hours. Skin injury was induced by tape stripping the depilated area six to twelve times with a cellophane tape (Scotch type). For stromal cell analysis with flow cytometry, mice were euthanized 18 hours after tape striping, and skin dissected and prepared as described above. For monitoring hair regrowth, mice were examined twice a week after skin abrasion, and pictures taken between day 12 and day 14 after tape stripping. Anagen induction was assessed visually. An approximately 1cm wide skin strip from the same location across all mice in a cage was dissected for immunofluorescent thick sections staining and the analysis of HF length.

#### DRG immunofluorescent staining and image quantification

7- to 8-week-old C57BL/6J mice had their entire back skin depilated 72 hours before the experiment to allow sensory acclimation. Then, half of the mice assigned for the experiment were randomly selected to be tape stripped ten times with cellophane tape, as described above. Control mice were subjected to the same handling procedure but not tape stripped. Exactly 90 minutes later, mice were perfused with PBS and then 4% PFA. DRGs were dissected and fixed in 4% paraformaldehyde at 4°C for 1 hour, cryoprotected in 30% sucrose in PBS for 24 hours and embedded in OCT at −80°C. DRG tissue were cut at 12–14 μm in a cryostat and mounted onto Superfrost plus slides. Primary antibodies used were rabbit anti-c-Fos (1:500) and guinea pig anti-TRPV1 (1:400). Secondary antibodies used were Alexa488-conjugated donkey anti-guinea pig IgG, Alexa647-conjugated goat anti-rabbit IgG. Slides were mounted with Fluoroshield^™^ with DAPI and imaged on a Zeiss AxioObserver Z1 inverted microscope with Apotome attachment.

### Quantification and statistical analysis

Statistical analyses were performed using GraphPad Prism software 7. Unpaired two-tailed Student’s t test was used to compare two groups. For multiple comparisons, one-way analysis of variance (ANOVA) with Bonferroni’s multiple comparisons test was used. P values smaller than 0.05 were considered statistically significant. Error bars represent mean ± standard error of the mean (SEM). Except for the scRNAseq experiments, c-Fos staining in DRG after tape stripping (done twice), Spp1 staining with flow cytometry 18 hours after tape stripping (done twice) and the monitoring of hair growth in Spp1 KO mice and their controls (done once), all experiments were performed at least 3 times independently. The Spp1 KO analysis provided confirmation of findings for the Spp1 blocking antibody treatment experiments that were done 3 times.

## Supplementary Material

2

## Figures and Tables

**Table:** Key resources table

REAGENT or RESOURCE	SOURCE	IDENTIFIER
Antibodies		
Percp-Cy5.5 anti-mouse CD45.2 (104)	BioLegend	Cat# 109828; RRID: AB_893350
FITC anti-mouse CD45.1 Antibody (A20)	BioLegend	Cat# 110706; RRID: AB_313495
Brilliant Violet 711 anti-mouse CD326 (Ep-CAM) (G.8)	BioLegend	Cat# 118233; RRID: AB_2632775
PerCP/Cyanine5.5 anti-mouse CD31 (MEC13.3)	BioLegend	Cat# 102522; RRID: AB_2566761
Brilliant Violet 785^™^ anti-mouse CD90.2 (Thy1.2) (30-H12)	BioLegend	Cat# 105331; RRID: AB_2562900
Brilliant Violet 605^™^ anti-mouse Ly-6A/E (Sca-1) (D7)	BioLegend	Cat# 108134; RRID: AB_2650926
PE/Cyanine7 anti-mouse CD26 (DPP-4) (H194-112)	BioLegend	Cat# 137810; RRID: AB_2564312
Biotin anti-mouse CD9 (MZ3)	BioLegend	Cat# 124804; RRID: AB_2275863
FITC anti-mouse I-A/I-E (M5/114.15.2)	BioLegend	Cat# 107606; RRID: AB_313321
PE/Cyanine7 anti-mouse CD11c (N418)	BioLegend	Cat# 117318; RRID: AB_493568
Brilliant Violet 605 anti-mouse Ly6C (HK1.4)	BioLegend	Cat# 128036; RRID: AB_2562353
Brilliant Violet 421 anti-mouse Ly-6G (1A8)	BioLegend	Cat# 127628; RRID: AB_2562567
Brilliant Violet 785 anti-mouse/human CD11b (M1/70)	BioLegend	Cat# 101243; RRID: AB_2561373
APC anti-mouse CD64 (FcγRI) (X54-5/7.1)	BioLegend	Cat# 139306; RRID: AB_11219391
PE anti-human/mouse CD49f (Itga6) (GoH3)	BioLegend	Cat# 313612; RRID: AB_893373
Brilliant Violet 605^™^ anti-mouse CD103 (2E7)	BioLegend	Cat# 121433; RRID: AB_2629724
APC anti-mouse/human CD207 (Langerin) (4C7)	BioLegend	Cat# 144206; RRID: AB_2561998
Biotin anti-βIII-tubulin (TUJ1)	BioLegend	Cat# 801212; RRID: AB_2721321
PE anti-mouse F4/80 (BM8)	BioLegend	Cat# 123110; RRID: AB_893486
APC anti-mouse CD172a (SIRPα) (P84)	BioLegend	Cat# 144014; RRID: AB_2564061
FITC anti-mouse Ly-6G/Ly-6C (Gr-1) (RB6-8C5)	BioLegend	Cat# 108406; RRID: AB_313371
Rabbit mAb anti-HA-Tag (C29F4)	Cell Signaling	Cat# #3724; RRID: AB_1549585
Alexa Fluor 647 Mouse anti-Ki67 (B56)	BD Biosciences	Cat# 558615; RRID: AB_647130
FITC mouse anti-Cytokeratin 14 (LL002)	Invitrogen	Cat# MA5-28125; RRID: AB_2745108
Goat anti-mouse Spp1 polyclonal	R&D Systems	Cat# AF808; RRID: AB_2194992
Fixable Viability Dye eFluor^™^ 780	Fisher Scientific	Cat# 65-0865-18
Rabbit Anti c-Fos (9F6)	Cell Signaling	Cat# 2250; RRID: AB_2247211
Alexa Flour 647 Goat anti-Rabbit IgG (H+L) Highly Cross-Adsorbed Ab	Invitrogen	Cat# A21245; RRID: AB_2535813
Alexa Flour 647 Donkey anti-Goat IgG (H+L) Highly Cross-Adsorbed Ab	Invitrogen	Cat# A-21447; RRID: AB_2535864
Alexa Fluor^®^ 488 AffiniPure^™^ Goat Anti-Guinea Pig IgG (H+L)	Jackson ImmunoResearch	Cat# 106-545-003; RRID: AB_2337438
Guinea pig anti-TRPV1	Invitrogen	Cat # PA1-29770; RRID: AB_1958678
TotalSeq^™^-A0301 anti-mouse Hashtag 1	BioLegend	Cat # 30-F11; RRID: AB_2750032
TotalSeq^™^-A0302 anti-mouse Hashtag 2	BioLegend	Cat #155803; RRID: AB_2750033
InVivoMAb anti-mouse Ly6G (1A8)	BioXCell	Cat # BE0075-1; RRID: AB_1107721
InVivoMAb Anti-Spp1 neutralizing antibody (100D3)	BioXCell	Cat # #BE0372; RRID: AB_2927509
InVivoMAb anti-mouse CSF1R (AFS98)	BioXCell	Cat # BE0213; RRID: AB_2687699
Chemicals, peptides, and recombinant proteins		
Liberase TM Research Grade	Roche/Sigma	Cat # LIBTM-RO
Deoxyribonuclease I from bovine pancreas	Sigma	Cat# DN25
Fluoroshield^™^ with DAPI	Sigma-Aldrich	Cat# F6057
hyaluronidase	Sigma-Aldrich	Cat# H3506
CGRP 8-37 (rat)	Tocris	Cat# 1169
QWF	Tocris	Cat# 6642
R428	APExBIO	Cat # A8329
Diphtheria Toxin (DT)	Calbiochem /Sigma	Cat# 322326
Clozapine N-oxide	Tocris	Cat# 4936
Critical commercial assays		
BD Cytofix/Cytoperm^™^ Fixation/Permeabilization Kit	BD Biosciences	Cat # 323100
RNAscope multiplex fluorescent detection kit v2	ACD	Cat # 323100
TSA Vivid Fluorophore Dyes 520	ACD	Cat # 323271
TSA Vivid Fluorophore Dyes 650	ACD	Cat # 323273
Spp1 probe	ACD	Cat # 435191
Lepr probe	ACD	Cat # 402731-C2
In Situ Cell Death Detection Kit, Fluorescein	Roche/Sigma	Cat # 11684795910
Clodrosome	Encapsula Nano Sciences	Cat # CLD-8909
Deposited data		
Raw data	Lie et al.^41^	GEO: GSE160513
Mouse 3’-single-cell RNA-seq data (sorted Lin- Thy1+ SCA1+).	This manuscript	GEO: GSE266028
Mouse 3’-single-cell RNA-seq data (sorted CD45+).	This manuscript	GEO: GSE266027
Experimental models: Organisms/strains		
C57BL/6J	Jackson	B6 background
BoyJ (CD45.1)	Jackson	B6 background
TRPV1Cre	Allen Basbaum, UCSF^84^	B6 background
B6N;129-Tg(CAG-CHRM3*,-mCitrine)1Ute/J	Jackson ^85^	B6 background
C57BL/6-Gt(ROSA)26Sortm1(HBEGF)Awai/J	Jackson^86^	B6 background
B6.129P2 Gt(ROSA)26Sortm1(DTA)Lky/J	Jackson ^87^	B6 background
B6.129(Cg)-Scn10atm2(cre)Jwo/TjpJ	Jackson ^75^	B6 background
B6.129X1-Twist2tm1.1(cre)Dor/J	Jeffery Bush, UCSF ^88^	B6 background
B6.129S2(Cg)-Ramp1tm1.1Tsuj/WkinJ	Jackson ^18^	B6 background
CD64Cre	Russell Vance, UC Berkeley ^89^	B6 background
B6.129S6(Cg)-Spp1tm1Blh/J	Jackson^46^	B6 background
Gnas floxed	Guo Huang, UCSF ^90^	B6 background
C57BL/ 6N-Ramp1<tm1c(EUCOMM)Wtsi>/H	Pierangelo Geppetti, University of Florence, Italy	B6 background
Oligonucleotides		
TGG CTA AGG ACC AAG ACC ATC CAA -Fwd (IL6 qPCR)	This paper	N/A
AAC GCA CTA GGT TTG CCG AGT AGA- Rev (IL6 qPCR)	This paper	N/A
TCTTCTGTCTACTGAACTTCGGGGT- Fwd (TNF qPCR)	This paper	N/A
GGCCATAGAACTGATGAGAGGG - Rev (TNF qPCR)	This paper	N/A
GACCTGGGCACCATCCAT- Fwd (TGFb1 qPCR)	This paper	N/A
CCGCACACAGCAGTTCTT- Rev (TGFb1 qPCR)	This paper	N/A
ATC CCT CAA AGC TCA GCG TGT C- Fwd (IL17 qPCR)	This paper	N/A
GGG TCT TCA TTG CGG TGG AGA G- Rev (IL17 qPCR)	This paper	N/A
TAT CCA GTG TGA AGA TGG TTG TG- Fwd (IL23 qPCR)	This paper	N/A
CAC TAA GGG CTC AGT CAG AGT TG- Rev (IL23 qPCR)	This paper	N/A
Oligonucleotides for Aurkb, Ccnb1, Ccnb2, Cdca2, Rad51, Prc1 and Cdk1 provided in Figure S3A.	This paper	N/A
Software and algorithms		
FlowJo (v.10.6.2)	FlowJo^™^Software	N/A
Prism (GraphPad 9.0.1)	GraphPad Software	N/A
CellChat R package (1.6.1)	Jin et al. ^67^	https://github.com/sqjin/CellChat
CellRanger (version 7.0.0)	Zheng et al.^91^	https://github.com/10XGenomics/cellranger
Seurat R package (4.0.4)	Hao et al. ^92^	https://github.com/satijalab/seurat
Scanpy (1.8.2)	Wolf et al. ^93^	https://github.com/theislab/scanpy

## Highlights:

- Activation of cutaneous TRPV1 innervations accelerated hair growth in naïve skin
- TRPV1 activation induced a rapid apoptosis of dermal macrophages via CGRP
- TRPV1 activation triggered the expansion of Spp1 expressing dermal fibroblasts
- TRPV1, CGRP and Spp1 expression control hair regrowth after skin abrasion
