# Supplementary material for: Dermal TRPV1 innervations engage a macrophage and fibroblast containing pathway to activate hair growth in mice
Source: Dev Cell. Author manuscript; Available in PMC 2024 Nov 6. (PMC11537826; doi:10.1016/j.devcel.2024.05.019)
Supplement: 2 [file NIHMS2004247-supplement-2.pdf]

Supplementary Figures

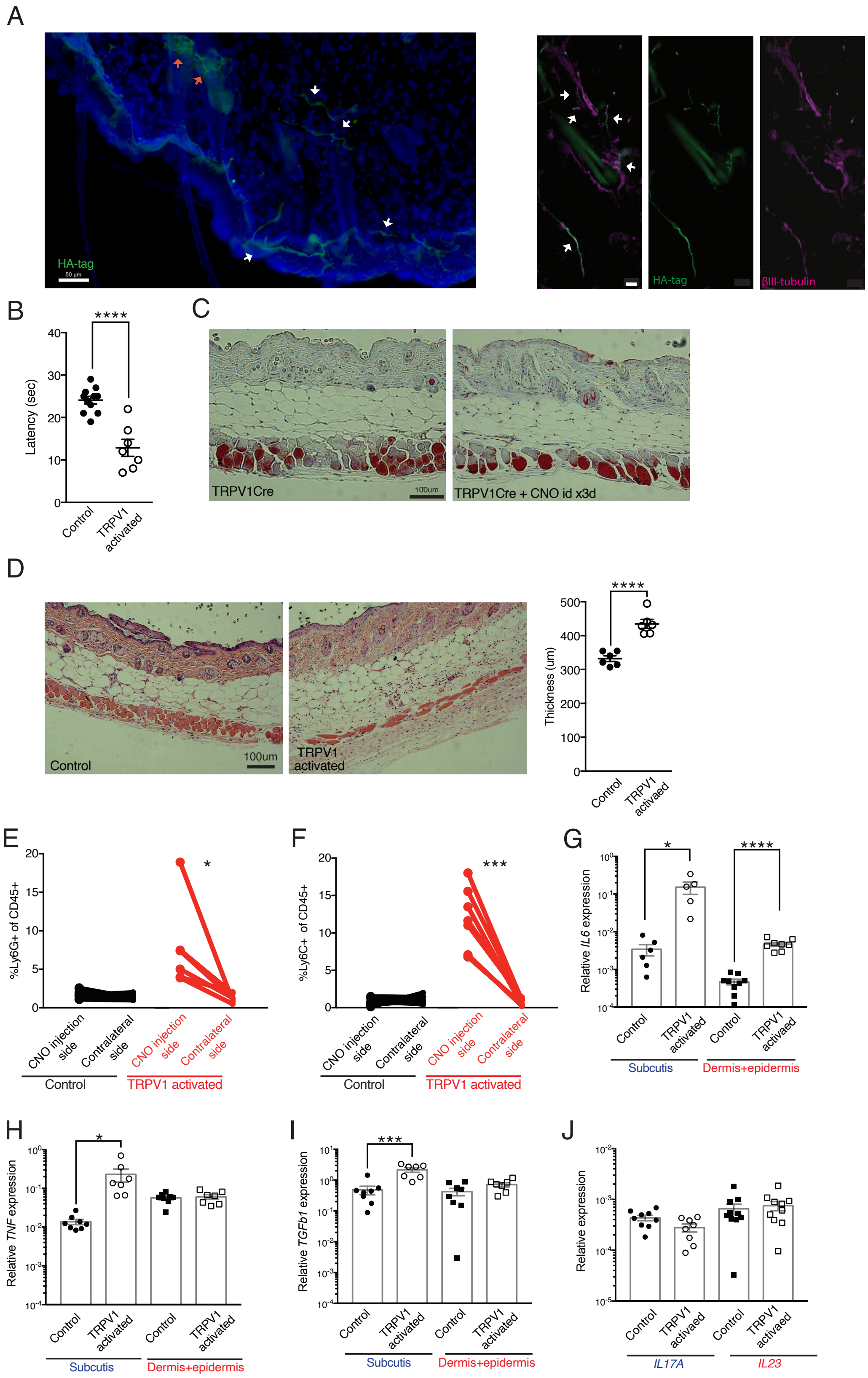

A
HA-tag
50 μm
HA-tag
βIII-tubulin
B
****
Latency (sec)
Control
TRPV1 activated
C
TRPV1Cre
100um
TRPV1Cre + CNO id x3d
D
Control
100um
TRPV1 activated
****
Thickness (um)
Control
TRPV1 activaed
E
*
%Ly6G+ of CD45+
CNO injection side
Contralateral side
Control
TRPV1 activated
F
***
%Ly6C+ of CD45+
CNO injection side
Contralateral side
Control
TRPV1 activated
G
*
****
Relative IL6 expression
Control
TRPV1 activated
Subcutis
Dermis+epidermis
H
*
Relative TNF expression
Control
TRPV1 activated
Subcutis
Dermis+epidermis
I
***
Relative TGFb1 expression
Control
TRPV1 activated
Subcutis
Dermis+epidermis
J
Relative expression
Control
TRPV1 activated
IL17A
IL23

Figure S1. Local DREADD-induced TRPV1 activation triggered skin inflammation (related to Figure 1).

(A) Representative images of 50μm naïve dorsal skin sections section from TRPV1Cre^{hM3Dq} mouse (Left) stained for HA-tag (green) and dapi (blue). Scale bar 50μm. (Right) stained for βIII-tubulin (purple) and HA-tag (green). White arrows indicate co-labelling. Scale bar 20μm.

(B) Effect of CNO injection in the footpad on response latencies on a hot plate in TRPV1 activated (TRPV1Cre ^{hM3Dq}) and control (TRPV1Cre) mice. ****p < 0.0001. Data shown as mean + SEM.

(C) Representative images of back skin sections from DREADD negative mice (TRPV1Cre) injected intradermally with CNO daily for three days, or untreated naïve skin. Bright field images labeled with trichrome staining. Scale bar 100μm.

(D) Representative images of skin sections from TRPV1 activated mice and their controls after three days of repeated intradermal injection of CNO. Bright field images labeled with H&E staining, and quantification of skin thickness above the Panniculus carnosus muscle. Scale bar 100μm. ****p < 0.0001. Data shown as mean + SEM.

(E) Neutrophils (CD45+ Epcam- CD11b+ Ly6G+ Ly6C-) and

(F) Monocytes (CD45+ Epcam- CD11b+ Ly6G- Ly6C+ F480-) collected from the back skin of TRPV1 activated mice (red lines) and their controls (black lines) after three days of repeated TRPV1 activation. For both experimental groups, the back skin site where CNO was inject was separated and compared to the contralateral side which was injected in the same manner with vehicle (PBS). *p < 0.042 (neutrophils) and ***p< 0.0003 (monocytes). Data shown as mean + SEM.

(G-J) Quantitative qPCR analysis of mRNA expression in the subcutis and the upper skin layers taken form the back of TRPV1 activated mice and their controls after three days of repeated CNO injection **(G)** *IL6,* *p< 0.015, ****p< 0.0001 **(H)** *TNF,* *p< 0.018 **(I)** *TGFb1*, ***p< 0.0007 and **(J)** expression of IL17A and IL23 in the upper skin layers only. Data shown as mean + SEM.

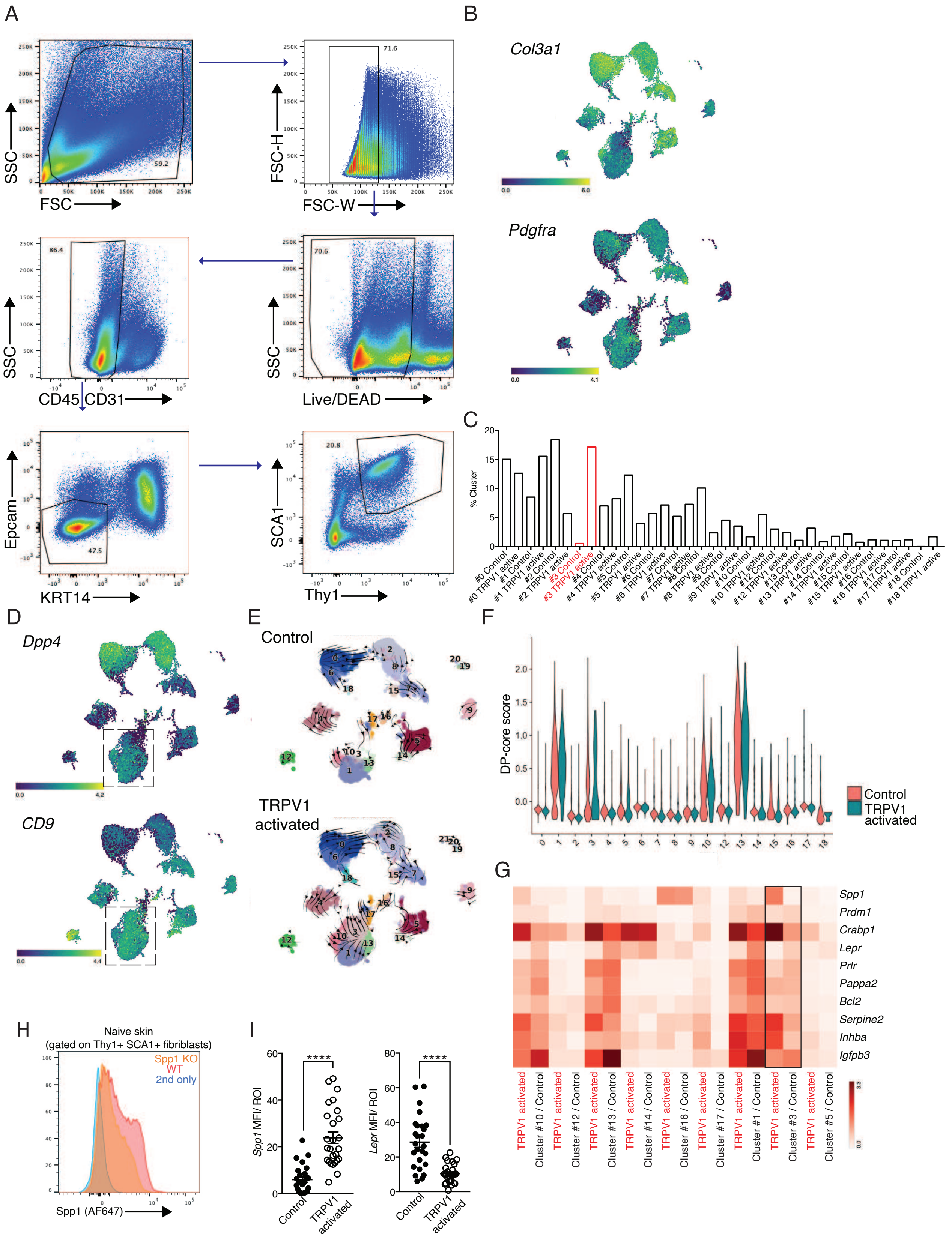
A
SSC
FSC
59.2
FSC-H
FSC-W
71.6
SSC
CD45 CD31
86.4
SSC
Live/DEAD
70.6
Epcam
KRT14
47.5
SCA1
Thy1
20.8
B
Col3a1
Pdgfra
C
% Cluster
#0 Control
#0 TRPV1 active
#1 Control
#1 TRPV1 active
#2 Control
#2 TRPV1 active
#3 Control
#3 TRPV1 active
#4 Control
#4 TRPV1 active
#5 Control
#5 TRPV1 active
#6 Control
#6 TRPV1 active
#7 Control
#7 TRPV1 active
#8 Control
#8 TRPV1 active
#9 Control
#9 TRPV1 active
#10 Control
#10 TRPV1 active
#12 Control
#12 TRPV1 active
#13 Control
#13 TRPV1 active
#14 Control
#14 TRPV1 active
#15 Control
#15 TRPV1 active
#16 Control
#16 TRPV1 active
#17 Control
#17 TRPV1 active
#18 Control
#18 TRPV1 active
D
Dpp4
CD9
E
Control
TRPV1
activated
F
DP-core score
Control
TRPV1
activated
G
Spp1
Prdm1
Crabp1
Lepr
Prlr
Pappa2
Bcl2
Serpine2
Inhba
Igfbp3
TRPV1 activated
Cluster #10 / Control
TRPV1 activated
Cluster #12 / Control
TRPV1 activated
Cluster #13 / Control
TRPV1 activated
Cluster #14 / Control
TRPV1 activated
Cluster #16 / Control
TRPV1 activated
Cluster #17 / Control
TRPV1 activated
Cluster #1 / Control
TRPV1 activated
Cluster #3 / Control
TRPV1 activated
Cluster #5 / Control
H
Naive skin
(gated on Thy1+ SCA1+ fibroblasts)
Spp1 KO
WT
2nd only
Spp1 (AF647)
I
Spp1 MFI/ ROI
****
Control
TRPV1
activated
Lepr MFI/ ROI
****
Control
TRPV1
activated

Figure S2. Dermal fibroblast gating strategy and gene expression patterns with scRNAseq (related to Figure 1).

(A) Gating strategy using flow cytometry identifying Thy1+ SCA1+ dermal fibroblasts.

(B) UMAP projection of sorted single cell fibroblasts demonstrating the expression of *Col3a1* and *Pdgfra.*

(C) Percent calculation comparing cell numbers in dermal clusters between TRPV1 activated mice and controls after three days of repeated CNO intradermal injection.

(D) UMAP projection of sorted single cell fibroblasts demonstrating the expression of *Dpp4* and *CD9.*

(E) RNA Velocity analysis overlaid on UMAP projection**.**

(F) Violin plot depicting DP-core scores across all clusters in the TRPV1 activated sample and control.

(G) Heat map visualizing gene expression (x-axis) across dermal clusters in the TRPV1 activated sample and control (y-axis). Color encodes average gene expression. Rectangle highlight cluster #3.

(H) Representative histogram showing anti-Spp1 staining with flow cytometry on dermal cells pre-gated on Lin- Thy1+ SCA1+ CD9+ CD26+ fibroblasts from the naïve skin of control wild type (orange) and Spp1 KO mouse (red). Secondary antibody background staining in blue.

(I) Quantification of *Spp1* and *Lepr* MFI signal in the lower follicle by RNAscope analysis. Data combined from 3-11 ROIs per mouse taken from 5 TRPV1 activated mice and 4 controls after three daily CNO intradermal injections. ****p < 0.0001. Data shown as mean + SEM.

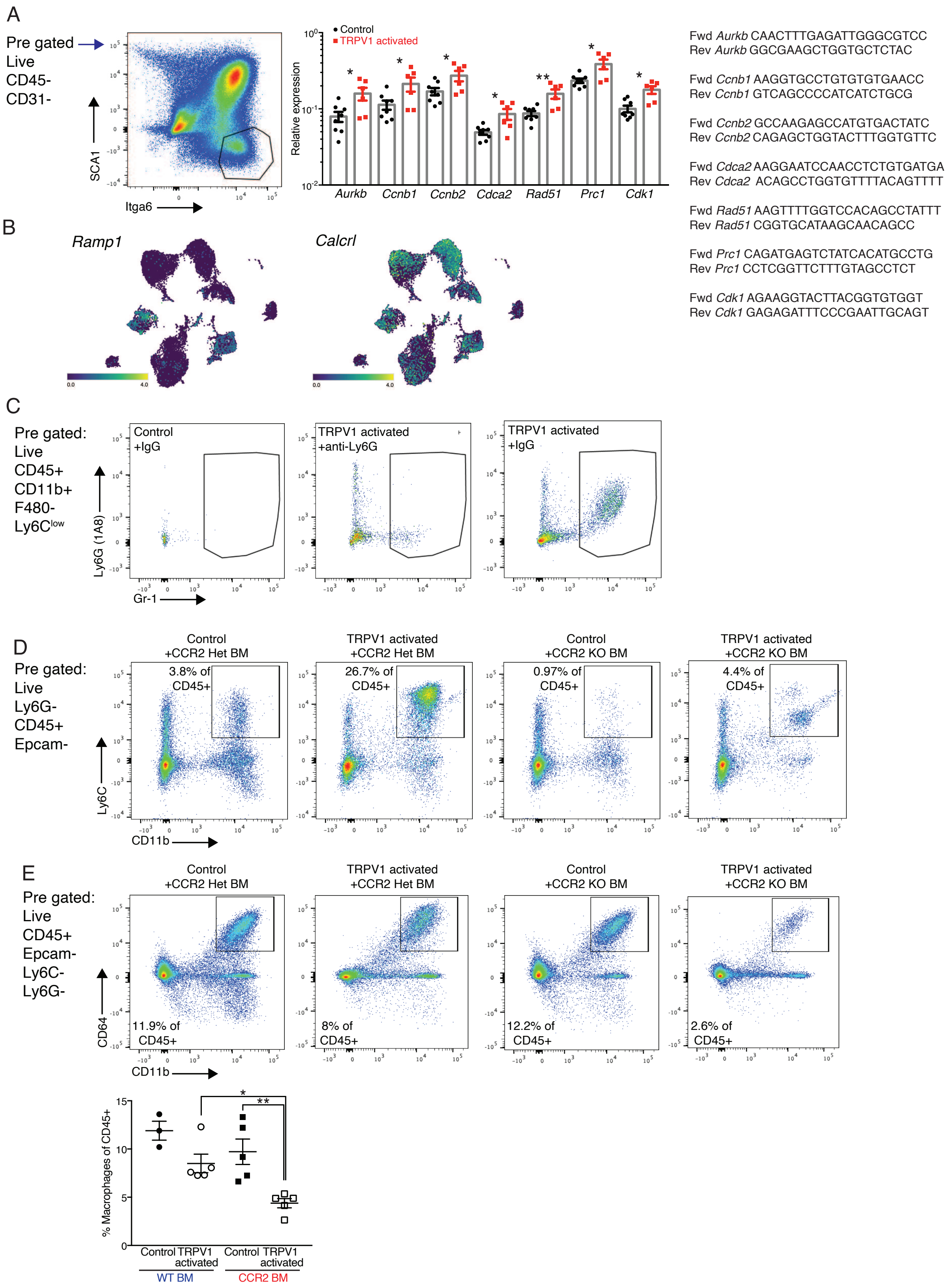

A
Pre gated
Live
CD45-
CD31-
SCA1
Itga6
Control
TRPV1 activated
Relative expression
Aurkb
Ccnb1
Ccnb2
Cdca2
Rad51
Prc1
Cdk1
Fwd Aurkb CAACTTTGAGATTGGGCGTCC
Rev Aurkb GGCGAAGCTGGTGCTCTAC
Fwd Ccnb1 AAGGTGCCTGTGTGTGAACC
Rev Ccnb1 GTCAGCCCCATCATCTGCG
Fwd Ccnb2 GCCAAGAGCCATGTGACTATC
Rev Ccnb2 CAGAGCTGGTACTTTGGTGTTC
Fwd Cdca2 AAGGAATCCAACCTCTGTGATGA
Rev Cdca2 ACAGCCTGGTGTTTTACAGTTTT
Fwd Rad51 AAGTTTTGGTCCACAGCCTATTT
Rev Rad51 CGGTGCATAAGCAACAGCC
Fwd Prc1 CAGATGAGTCTATCACATGCCTG
Rev Prc1 CCTCGGTTCTTTGTAGCCTCT
Fwd Cdk1 AGAAGGTACTTACGGTGTGGT
Rev Cdk1 GAGAGATTTCCCGAATTGCAGT
B
Ramp1
Calcrl
C
Pre gated:
Live
CD45+
CD11b+
F480-
Ly6Clow
Control
+IgG
TRPV1 activated
+anti-Ly6G
TRPV1 activated
+IgG
Ly6G (1A8)
Gr-1
D
Pre gated:
Live
Ly6G-
CD45+
Epcam-
Control
+CCR2 Het BM
TRPV1 activated
+CCR2 Het BM
Control
+CCR2 KO BM
TRPV1 activated
+CCR2 KO BM
3.8% of
CD45+
26.7% of
CD45+
0.97% of
CD45+
4.4% of
CD45+
Ly6C
CD11b
E
Pre gated:
Live
CD45+
Epcam-
Ly6C-
Ly6G-
Control
+CCR2 Het BM
TRPV1 activated
+CCR2 Het BM
Control
+CCR2 KO BM
TRPV1 activated
+CCR2 KO BM
11.9% of
CD45+
8% of
CD45+
12.2% of
CD45+
2.6% of
CD45+
CD64
CD11b
% Macrophages of CD45+
Control
TRPV1
activated
Control
TRPV1
activated
WT BM
CCR2 BM

Figure S3. qPCR analysis of cell cycle related genes in HFSC and the ablation efficiency in the skin of neutrophil and monocytes (related to Figure 2 and 3).

(A) Gating strategy for sorting HFSC (based on Choi S. et al[1] and Wang E. et al[2]) and qPCR analysis of genes related to cell cycle machineries and cytokinesis, using cells from TRPV1 activated mice and controls taken at day 7 after the last CNO injection. Oligonucleotides sequence for qPCR analysis. $*p < 0.05$, $**p<0.005$. Data shown as mean + SEM.

(B) UMAP projection of sorted single cell fibroblasts demonstrating the expression of *Ramp1* and *Calcrl.*

(C) Flow cytometry analysis demonstrating the efficiency of neutrophil ablation in the dermis with anti-Ly6G intraperitoneal injection given 24 hours before the first CNO treatment. Markers used for pre-gating and dot plots presented are from the dorsal skin of control mice given isotype control and TRPV1 activated mice given either anti-Ly6G or isotype control prior to initiating the three-day intradermal CNO injection protocol.

(D) and **(E)** Flow cytometry analysis demonstrating the effects of transferring CCR2 KO or heterozygous bone marrow to irradiated TRPV1 activated mice and controls. Eight weeks after bone marrow transfer, chimeric mice were given three daily intradermal CNO injections prior to acquiring data, **(D)** pre-gated CD45+ Epcam- dermal cells

(E) pre-gated CD45+ Epcam- Ly6C- Ly6G- dermal cells. Quantification of macrophage percentage in chimeric TRPV1 activated mice and controls. $**p=0.003$,$*=0.017$. Data shown as mean + SEM.

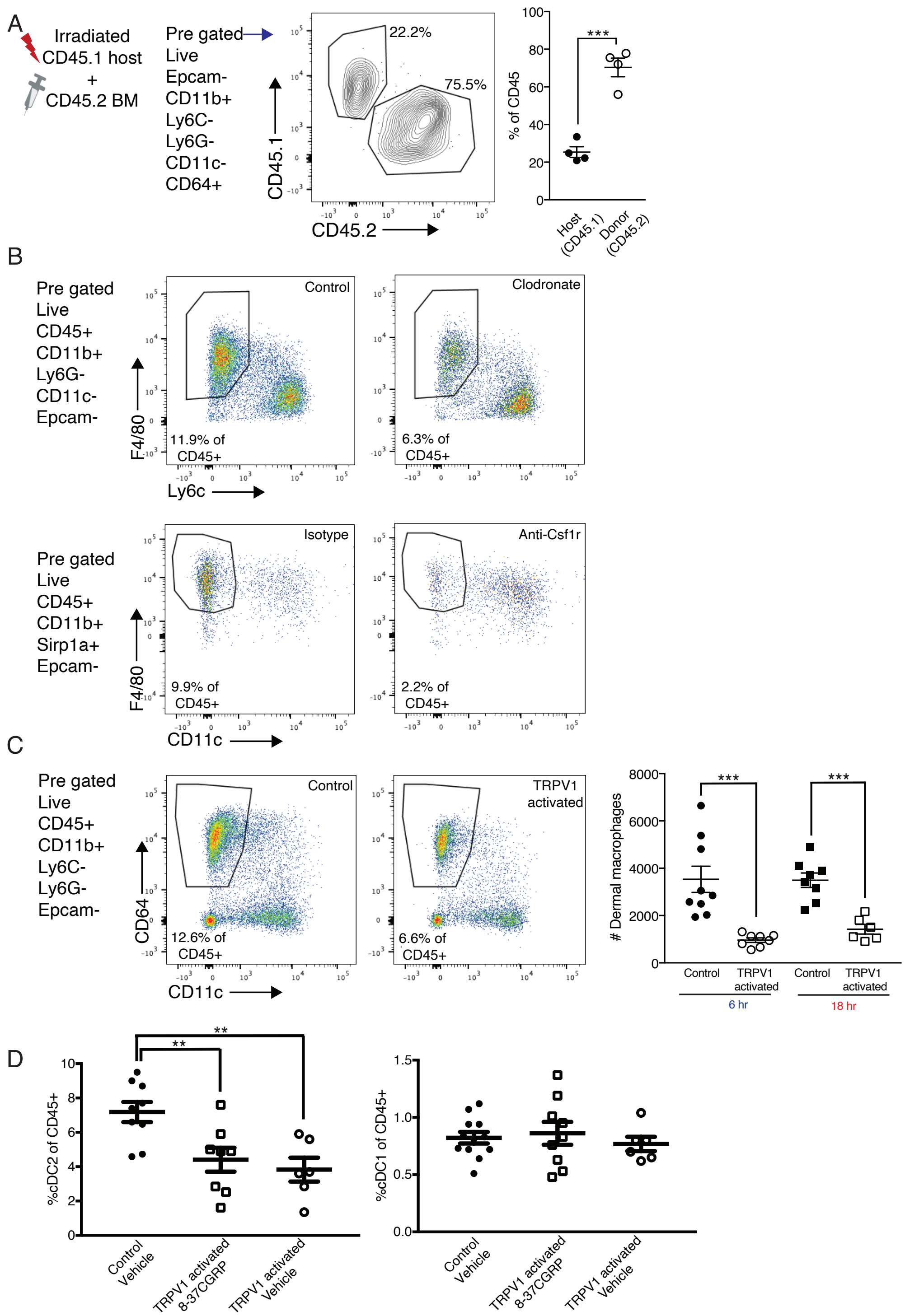

Figure S4. Dermal Macrophage chimerism and their ablation efficiency in the skin (related to Figure 3).

(A) Representative flow analysis, gating markers and quantification of chimerism efficiency for dermal macrophages. CD45.1 mice were irradiated and given bone marrow from CD45.2 donors.

Naïve back skin samples were collected 8 weeks later. ***$p < 0.0002$. Data shown as mean + SEM.

(B) Representative flow cytometry dot plot demonstrating the efficiency of systemic Clodronate liposomes and anti-Csf1r administration in ablating macrophages in naïve dorsal skin. Data presented shows the markers used for pre-gating and percentages calculated out of total dermal CD45+ immune cells.

(C) Representative flow cytometry dot plots showing staining for CD64 and CD11c and markers used for pre-gating on samples acquired from TRPV1 activated and control mice 6 hours after a single CNO intradermal injection. Quantification done on samples acquired 6 and 18 hours after a single CNO intradermal injection.

(D) Percentages of dermal cDC2 and cDC1 from TRPV1 activated and control mice 6 hours after a single CNO intradermal injection. **$p = 0.016, 0.007$. Data shown as mean + SEM.

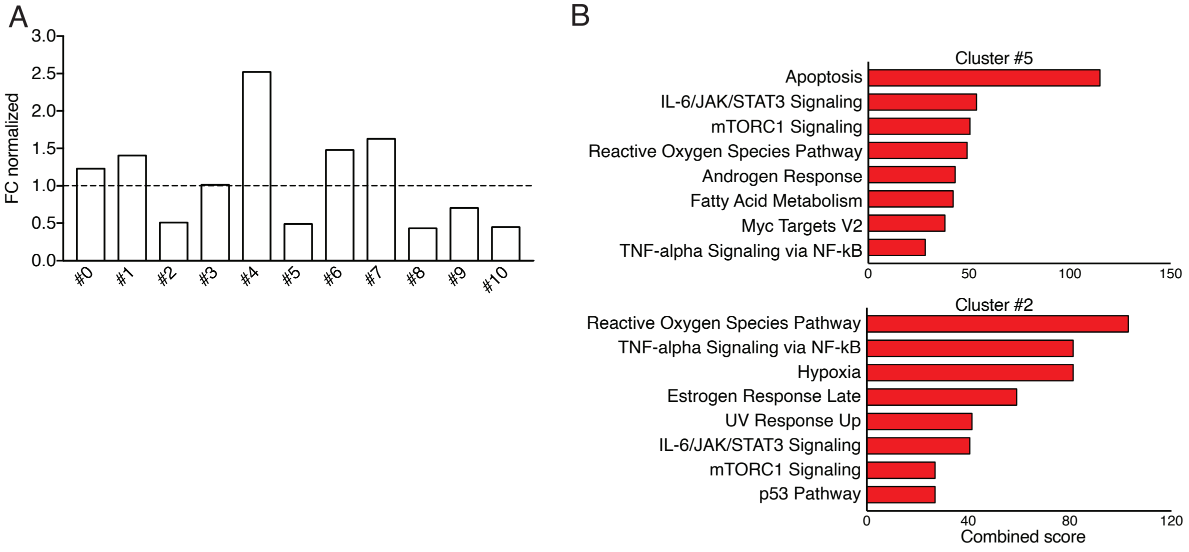

Figure S5. Dermal CD45+ cells scRNAseq cluster size and pathway analysis (related to Figure 5).

(A) Normalized fold change (FC) calculation comparing cluster's cell numbers between TRPV1 activated mice and their controls 3.5 hours after a single TRPV1 activation.

(B) Enricher pathway analysis for cluster 5 (Mertk+Fcgr1+ macrophages) and cluster #2 (cDC2) of differentially expressed genes (with adjusted p value lower then 1), between control and TRPV1 activated samples.

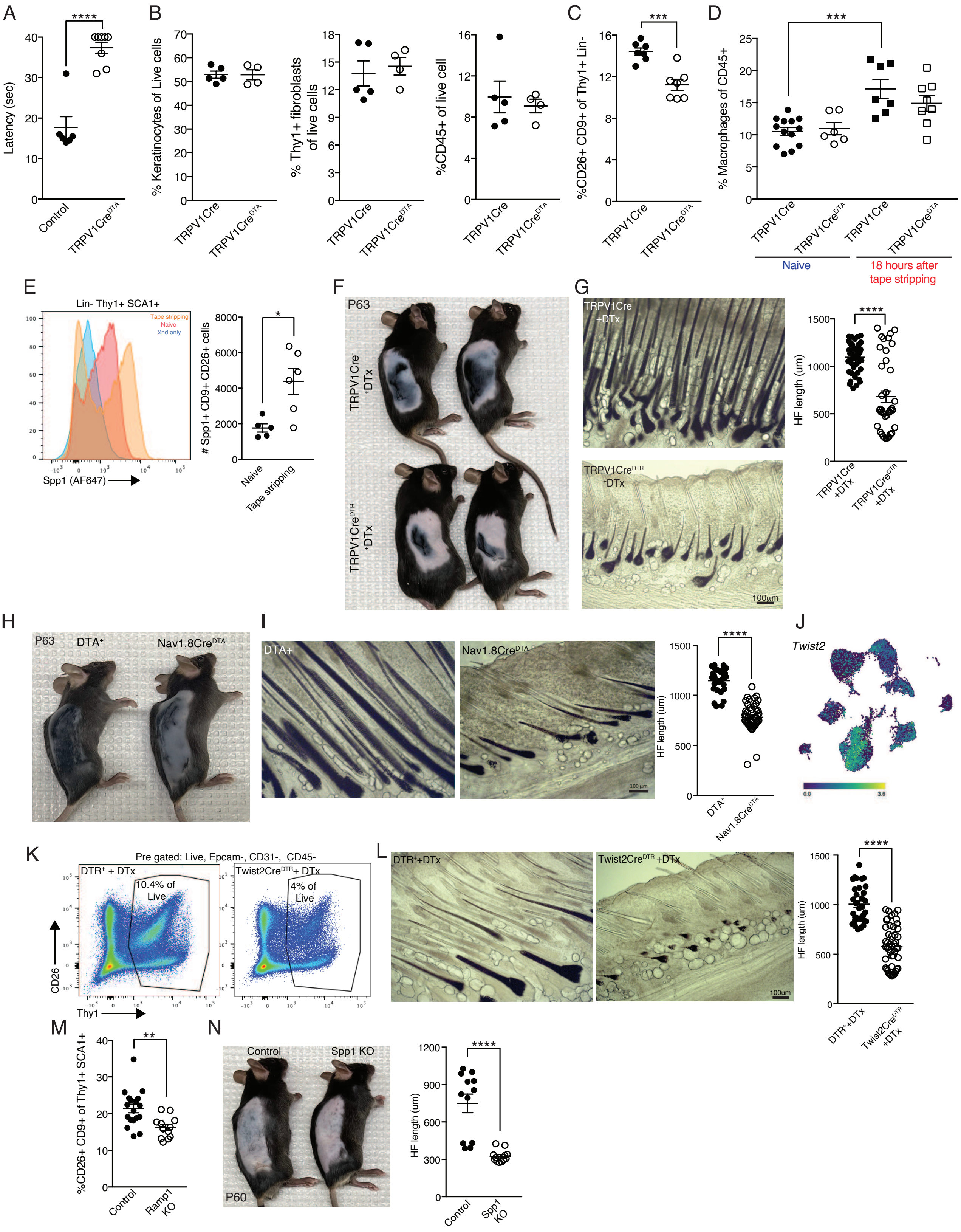
A
Latency (sec)
Control
TRPV1Cre^DTA
B
% Keratinocytes of Live cells
% Thy1+ fibroblasts of live cells
%CD45+ of live cell
TRPV1Cre
C
%CD26+ CD9+ of Thy1+ Lin-
D
% Macrophages of CD45+
Naive
18 hours after tape stripping
E
Lin- Thy1+ SCA1+
Tape stripping
Naive
2nd only
Spp1 (AF647)
Spp1+ CD9+ CD26+ cells
Tape stripping
F
P63
TRPV1Cre- +DTx
TRPV1Cre^DTR +DTx
G
TRPV1Cre +DTx
TRPV1Cre^DTR +DTx
100um
HF length (um)
H
P63
DTA+
Nav1.8Cre^DTA
I
DTA+
Nav1.8Cre^DTA
J
Twist2
K
Pre gated: Live, Epcam-, CD31-, CD45-
DTR+ + DTx
10.4% of Live
Twist2Cre^DTR+ DTx
4% of Live
CD26
Thy1
L
DTR+ +DTx
Twist2Cre^DTR +DTx
M
%CD26+ CD9+ of Thy1+ SCA1+
Control
Ramp1 KO
N
Control
Spp1 KO
P60

Figure S6. The effects on TRPV1, Twist2, Nav1.8, Ramp1 and Spp1 ablation of post epidermal abrasion hair growth (related to Figure 6).

(A) Response latencies in the hot plate test of TRPV1 genetic ablation ($TRPV1Cre^{DTA}$) and controls (TRPV1Cre). ****p < 0.0001. Data shown as mean ± SEM.

(B) Frequencies of keratinocytes (CD45- CD31- cytokeratin14+ Epcam+), dermal fibroblasts (CD45- CD31- Epcam- Thy1+) and leukocytes (CD45+ CD31- cytokeratin14-) from naïve dorsal skin of genetically TRPV1 ablated mice ($TRPV1Cre^{DTA}$) and controls (TRPV1Cre).

(C) Percentage of dermal CD9+ CD26+ fibroblasts from the naïve skin of TRPV1 ablated mice ($TRPV1Cre^{DTA}$) and their controls (TRPV1Cre) at telogen phase (7 weeks old). ***p < 0.001. Data shown as mean ± SEM.

(D) Percentage of dermal macrophages (CD45+ CD11b+ Ly6G- Ly6C- CD11c- CD64+) from naïve back skin and 18 hours after tape stripping collected from TRPV1 ablated (Trpv1CreDTA) and controls (Trpv1Cre). ***p< 0.0002. Data shown as mean ± SEM.

(E) Representative flow cytometry histograms of anti-Spp1 intracellular staining. Dermal cells from naive back skin (red) and 18 hours after tape stripping (orange) gated on Lin- Thy1+ and SCA1+ cells. Secondary only staining control in blue. Quantification of Spp1+ CD9+ CD26+ dermal fibroblasts from naïve back skin and after tape stripping. *p< 0.012. Data shown as mean ± SEM.

(F) Seven-week-old TRPV1 ablated mice ($TRPV1Cre^{DTR}$) and controls (TRPV1Cre) were pretreated with DTx prior to depilation and tape stripping. Picture taken 14 days after tape striping (postnatal day 63).

(G) Representative bright field images from dorsal skin samples at day 14 after tape stripping collected from TRPV1 ablated mice ($TRPV1Cre^{DTR}$) and controls (TRPV1Cre) pre-treated with DTx. HF length quantification was done at day 14 after tape stripping. Data collected from 5 TRPV1 ablated mice and 6 controls with 8 HFs analyzed per mouse. ****p < 0.0001. Data shown as mean ± SEM.

(H) Seven-week-old Nav1.8 ablated ($Nav1.8Cre^{DTA}$) and control (Nav1.8-Cre negative DTA positive) mice were depilated and tape stripped. Mice were monitored for hair coat recovery. Picture taken 14 days after tape striping (postnatal day 63).

(I) Representative bright field images of samples from Nav1.8 ablated mice ($Nav1.8Cre^{DTA}$) and controls (DTA^{+}) after dorsal skin depilation and tape stripping. Samples collected 14 days after

tape stripping. HF length quantification at day 14 after tape stripping collected from Nav1.8 ablated mice (Nav1.8Cre^{DTA}) and controls (DTA^{+}). Data consists of 8 HFs per mouse taken from 4 control and 6 Nav1.8+DTA+ mice. ****p < 0.0001. Data shown as mean ± SEM.

(J) UMAP projection of sorted single cell fibroblasts demonstrating the expression of *Twist2*.

(K) Representative flow dot plots showing the expression of Thy1 and CD26 on pre-gated Epcam- CD31- CD45- cells from dorsal skin of Twist2Cre^{DTR} mice and controls (DTR^{+}) after local intradermal injection of DTx for two days prior to acquiring samples.

(L) Representative bright field images and HF length quantification of samples from Twist2Cre^{DTR} mice and controls (DTR^{+}) treated locally with DTx before dorsal skin depilation and tape stripping. Picture taken at day 14 after tape stripping.

(M) Quantification of the percentage of CD9+ and CD26+ dermal fibroblasts from Lin- Thy1+ SCA1+ dorsal skin cells isolated from 7 weeks old Ramp1 KO mice and controls (C57BL/6) 18 hours after tape stripping. **p < 0.003. Data shown as mean ± SEM.

(N) Seven-week-old Spp1 KO and control (C57BL/6) mice were depilated, and tape stripped. Mice were monitored for hair coat recovery. Picture taken 11 days after tape striping (postnatal day 60). HF length quantification at day 11 after tape stripping with data combined from 4 HFs per mouse taken from 3 control and 3 Spp1 KO mice. ****p < 0.0001. Data shown as mean ± SEM.

Supplementary references

1. Choi, S., Zhang, B., Ma, S., Gonzalez-Celeiro, M., Stein, D., Jin, X., Kim, S.T., Kang, Y.L., Besnard, A., Rezza, A., et al. (2021). Corticosterone inhibits GAS6 to govern hair follicle stem-cell quiescence. Nature *592*, 428-432.
2. Wang, E.C.E., Dai, Z., Ferrante, A.W., Drake, C.G., and Christiano, A.M. (2019). A Subset of TREM2(+) Dermal Macrophages Secretes Oncostatin M to Maintain Hair Follicle Stem Cell Quiescence and Inhibit Hair Growth. Cell Stem Cell *24*, 654-669.
